# Tec1 Mediates the Pheromone Response of the White Phenotype of *Candida albicans*: Insights into the Evolution of New Signal Transduction Pathways

**DOI:** 10.1371/journal.pbio.1000363

**Published:** 2010-05-04

**Authors:** Nidhi Sahni, Song Yi, Karla J. Daniels, Guanghua Huang, Thyagarajan Srikantha, David R. Soll

**Affiliations:** Department of Biology, The University of Iowa, Iowa City, Iowa, United States of America; David Geffen School of Medicine at University of California Los Angeles, United States of America

## Abstract

The way in which signal transduction pathways evolve remains a mystery, primarily because we have few examples of ones that have newly evolved. There are numerous examples of how signal transduction pathways in the same organism selectively share components, most notably between the signal transduction pathways in *Saccharomyces cerevisiae* for the mating process, the filamentation process, cell wall integrity, ascospore formation, and osmoregulation. These examples, however, have not provided insights into how such pathways evolve. Here, through construction of an overexpression library for 107 transcription factors, and through mutational analyses, we have identified the transcription factor Tec1 as the last component of the newly evolved signal transduction pathway that regulates the pheromone response of the white cell phenotype in *Candida albicans*. The elucidation of this last component, Tec1, establishes a comprehensive description of the pheromone response pathway in the white cell phenotype of *C. albicans*, providing a unique perspective on how new signal transduction pathways may evolve. The three portions of this new regulatory pathway appear to have been derived from three different ancestral programs still functional in *C. albicans*. The upstream portion, including signals, receptors, the trimeric G protein complex, and the MAP kinase cascade, was derived intact from the upstream portion of the opaque pheromone response pathway of the mating process; Tec1, the transcription factor targeted by the MAP kinase pathway, was derived from a filamentation pathway; and the white-specific downstream target genes were derived from an ancestral biofilm process. The evolution of this pheromone response pathway provides a possible paradigm for how such signal transduction pathways evolve.

## Abstract 2

The newly evolved pheromone response pathway of the white cell phenotype of the opportunistic human pathogen *Candida albicans* provides a unique view of how signal transduction pathways evolve.

## Author Summary

Signal transduction pathways regulate the response of cells to changes in the extracellular environment. Here, we report the identification of Tec1 as the single effector transcription factor of the pheromone response pathway of the human pathogen *Candida albicans* white cell type. This newly evolved pathway provides us with a unique opportunity to investigate signal transduction pathway evolution. In the *C. albicans* white-opaque transition, mating-competent opaque cells release mating pheromone that induces mating-incompetent white cells to form a biofilm which facilitates mating of the former. Each of the three major portions of the pathway that regulates white cell pheromone response appears to be derived from an ancestral pathway that is still intact and functional in *C. albicans*. The upstream portion—including the pheromone, its receptor, trimeric G protein complex, and a MAP kinase cascade—appears to be derived from the mating response pathway; transcription factor Tec1 from the filamentation pathway; and Tec1 target genes from the biofilm biosynthesis pathway. We posit that the sharing of upstream signaling components coordinates white and opaque cell pheromone responses, yet the divergence of downstream pathway components allows each cell type to elicit a unique phenotypic outcome. The white cell pheromone response pathway therefore provides a paradigm for how other such pathways may have evolved.

## Introduction

There are numerous examples of how signal transduction pathways within an organism selectively share components [1]–[5]. For instance, in the yeast *Saccharomyces cerevisiae*, components are shared to varying degrees among pathways regulating mating, filamentation, cell wall integrity, ascospore formation, and osmoregulation [6]–[10]. However, such examples have not engendered a discussion of how signal transduction pathways evolve. For such insights, one must identify and interpret the origins of components in very new, or “young,” signal transduction pathways. Here, the elucidation of the last unidentified component of the pheromone response pathway of the white phenotype of *Candida albicans*, an opportunistic human fungal pathogen, provides such a view, which may prove to be a paradigm for how some signal transduction pathways evolve.

In order for *C. albicans* to mate, it must undergo homozygosis from **a**/α to **a**/**a** or α/α [11]–[13], then switch from the white to opaque phenotype [14],[15]. This latter requirement appears to be unique to *C. albicans* and the highly related species *Candida dubliniensis* [16]. The reason for this dependency may be due in part to a unique signaling system that evolved between opaque and white cells that leads to the formation of a biofilm that can facilitate mating between minority opaque cells [17],[18]. Mating-competent opaque cells, but not mating-incompetent white cells, release pheromone that induces a cell cycle block in G1, polarization, shmoo formation, and the expression of a number of mating-associated genes in opaque cells of opposite mating type [17],[19],[20]. Pheromone released by opaque cells has none of these effects on white cells of opposite mating types. Pheromone does, however, induce in these cells adhesion, cohesion, enhancement of biofilm formation, and the expression of select genes involved in these responses [10],[17],[21]–[23]. White cell biofilms have been shown in vitro to facilitate chemotropism between minority opaque cells of opposite mating types [17], leading to the hypothesis that the white-opaque switching system evolved from the direct ancestor of *C .albicans* and *C. dubliniensis* to facilitate mating [18].

The white cell pheromone response is mediated by a signal transduction pathway that shares all of the upstream components with the opaque cell signal transduction pathway involved in mating, including the pheromone signal, the pheromone receptors, the heterotrimeric G protein complex, and the mitogen-activated protein (MAP) kinase cascade [10]. The pheromone response pathway in opaque cells targeted the transcription factor Cph1, an ortholog of the *S. cerevisiae* transcription factor Ste12 [24],[25], which activates mating-associated genes [10],[23],[26],[27]. The pheromone response pathway in white cells, however, was found not to target Cph1, but rather an unknown transcription factor [10]. Cph1 up-regulated genes through the common GC-rich *cis*-acting sequence OPRE, whereas the unidentified white-specific transcription factor up-regulated genes through the common AT-rich *cis*-acting sequence WPRE [23]. Many of the genes that were found to be up-regulated by the white-specific transcription factor, but were not components of the pheromone response pathway, had already been implicated directly or indirectly in biofilm formation in **a**/α cells and, through mutational analyses, were shown to be involved in pheromone-induced white cell biofilm formation [23]. These observations have led to a unique glimpse into the evolution of a relatively new signal transduction pathway, the white cell pheromone response pathway [23], which is probably no older than the common ancestor of *C. albicans* and *C. dubliniensis*, given that white-opaque switching is unique to these two highly related species. It appears that in the evolution of the white pheromone response pathway, the upstream portion was borrowed intact from the mating pathway conserved in the Hemiascomycetes [28],[29], whereas the downstream target genes appeared to have been borrowed from the presumably conserved biofilm process of **a**/α cells. Only one piece of the pathway was missing, namely the identity and origin of the transcription factor connecting the upstream portion of the pathway and the downstream target genes.

To identify this factor, we generated an overexpression library of 107 individual **a**/**a** strains, each transformed with a construct in which a different transcription factor was placed under the control of a tetracycline-inducible promoter [30]. A screen for the unidentified white-specific transcription factor was then performed based on the assumption that overexpression of the correct transcription factor in the absence of pheromone would induce the white cell pheromone response. Using the dramatic increase in adhesion associated with the white cell pheromone response [17] as an assay, we found that only one of the 107 transcription factors, when overexpressed in the absence of pheromone, induced adhesion. The factor was Tec1, which had previously been shown to play a role in filamentation in both *S. cerevisiae* [31]–[33] and *C. albicans* [34]. Characterization of the *TEC1* deletion mutant *tec1/tec1* confirmed that it encoded the target transcription factor in the white cell pheromone response pathway. With this last component of the pathway identified, the evolution of the white-specific pheromone response pathway suggested a scenario in which all major components of the pathway had been derived from three conserved ancestral programs still functioning in *C. albicans*, providing a possible paradigm for how new signal transduction pathways may evolve.

## Results

### Generating an Overexpression Library for Transcription Factors

Our strategy to identify the transcription factor targeted by the MAP kinase pathway involved a screen in which the overexpression strains in the library were tested for the white cell response in the absence of pheromone. To accomplish this, we first identified 107 putative transcription factor genes involved in biofilm formation, adhesion, filamentation, cell wall integrity, membrane biogenesis, drug resistance, stress responses, and metabolism, and in a few cases of unknown function (Table S1). Each gene was synthesized by the polymerase chain reaction using the primers listed in Table S2, verified by sequencing and inserted into the expression site of the plasmid pNIM1 under the control of a promoter inducible by tetracycline or doxycycline [30], which we will refer to as *TETp*. This promoter was derived from the *tet* operator regulating tetracycline-resistant genes in *Escherichia coli* [30]. Each transcription factor gene was fused in frame at the 3′ end with the GFP ORF. Each of the 107 plasmids was then used to transform the natural **a**/**a** strain P37005 by integration at the *ADH1* locus, generating an overexpression library (Table S3).

### Screen for the White-Specific Pheromone-Induced Transcription Factor

**Figure 1:**
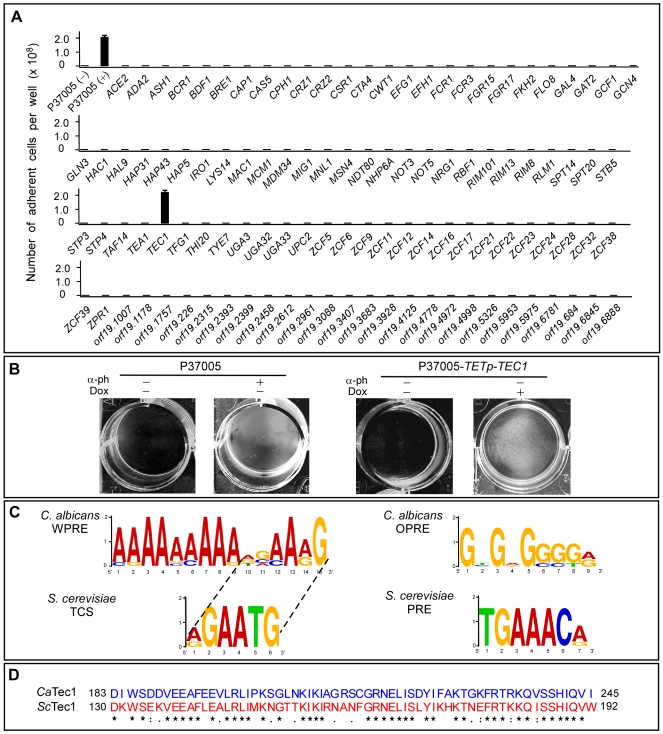
Screening of an overexpression library of 107 putative transcription factor genes of *Candida albicans*. This screening process revealed that overexpression of only one gene, *TEC1*, in the absence of α-pheromone, induced more than a 100-fold increase in adhesion. (A) Number of adherent cells per well bottom for the 107 strains in the presence of doxycycline and the absence of α-pheromone. For comparison, the levels in the control strain P37005 are also presented both in the absence (−) and presence (+) of α-pheromone. (B) Examples of adhesion of P37005 and the overexpression P37005-*TETp*-*TEC1* in the absence (−) and presence (+) of α-pheromone (α-ph) or doxycycline (Dox). (C) A comparison of WPRE and TCS, the putative *cis*-acting sequences interacting with Tec1 in *C. albicans* and *S. cerevisiae*, respectively, and OPRE and PRE, interacting with Cph1 and the homolog Ste12 in *C. albicans* and *S. cerevisiae*, respectively. (D) Comparison of the Tec1 DNA-binding domains of *C. albicans* (*Ca*) and *S. cerevisiae* (*Sc*).

Each of the 107 overexpression strains was tested in the absence of pheromone for increased adhesion to a plastic surface in the absence or presence of 100 µg per ml of doxycycline. To obtain a measure of maximum pheromone induction, white cells of the parental control strain P37005 were first analyzed in the absence and presence of α-pheromone. Adhesion was negligible (<10^6^ cells per well bottom) in the absence and maximal (>10^8^ cells per well bottom) in the presence of α-pheromone (Figure 1A). In the absence of α-pheromone, doxycycline induced adhesion in only one of the 107 overexpression strains, and did so to the same extent as α-pheromone induction in control cells (Figure 1A). That single strain overexpressed the gene *TEC1*. In Figure 1B, induction in the *TEC1* overexpression strain is obvious in images of the well bottoms after rinsing. *TEC1* has been shown to be involved in the induction of filamentation in both *S. cerevisiae* [32],[35] and *C. albicans* [34], as well as during biofilm formation in *C. albicans* **a**/α strains [36]. It should be noted that overexpression of *CPH1*, the transcription factor mediating the opaque pheromone response [10],[26],[27], had no effect on adhesion (Figure 1A).

In *S. cerevisiae*, Tec1 binds to the *cis*-acting AT-rich motif, TCS (TEA consensus sequence), in the promoters of Tec1-regulated genes (Figure 1C) [31],[37]. In *C. albicans*, WPRE (the white pheromone response element), the presumed Tec1 binding site is also AT-rich, in contrast to the Cph1 binding site, which is GC-rich (Figure 1C) [23]. The genes used to generate consensus motifs for *cis*-acting sequences with the MEME program [38]–[40] are presented in Table S4. Although the MEME program identified a consensus sequence, WPRE, which was 15 bases in length in *C. albicans*, that was longer than the TCS consensus sequence in *S. cerevisiae*, WPRE contains nearly intact the TCS motif as a subcomponent. This WPRE subcomponent differs from TCS by only a single base mismatch (Figure 1C). This *cis*-acting sequence has been conserved to some extent across species, including humans (Table S5).

The DNA binding domain of Tec1 in *C. albicans* is 63 amino acids in length (amino acids 183 to 245), whereas the deduced binding domain of Tec1 in *S. cerevisiae* is 413 amino acids (amino acids 74 to 486). The *C. albicans* binding domain shows strong homology (60% identity, 84% similarity) to a subdomain of the *S. cerevisiae* binding domain, between amino acids 130 and 192 (Figure 1D). The DNA binding domain of Tec1 is highly conserved in the ascomycetes (Figure S1).

### Regulation of *TEC1*

**Figure 2:**
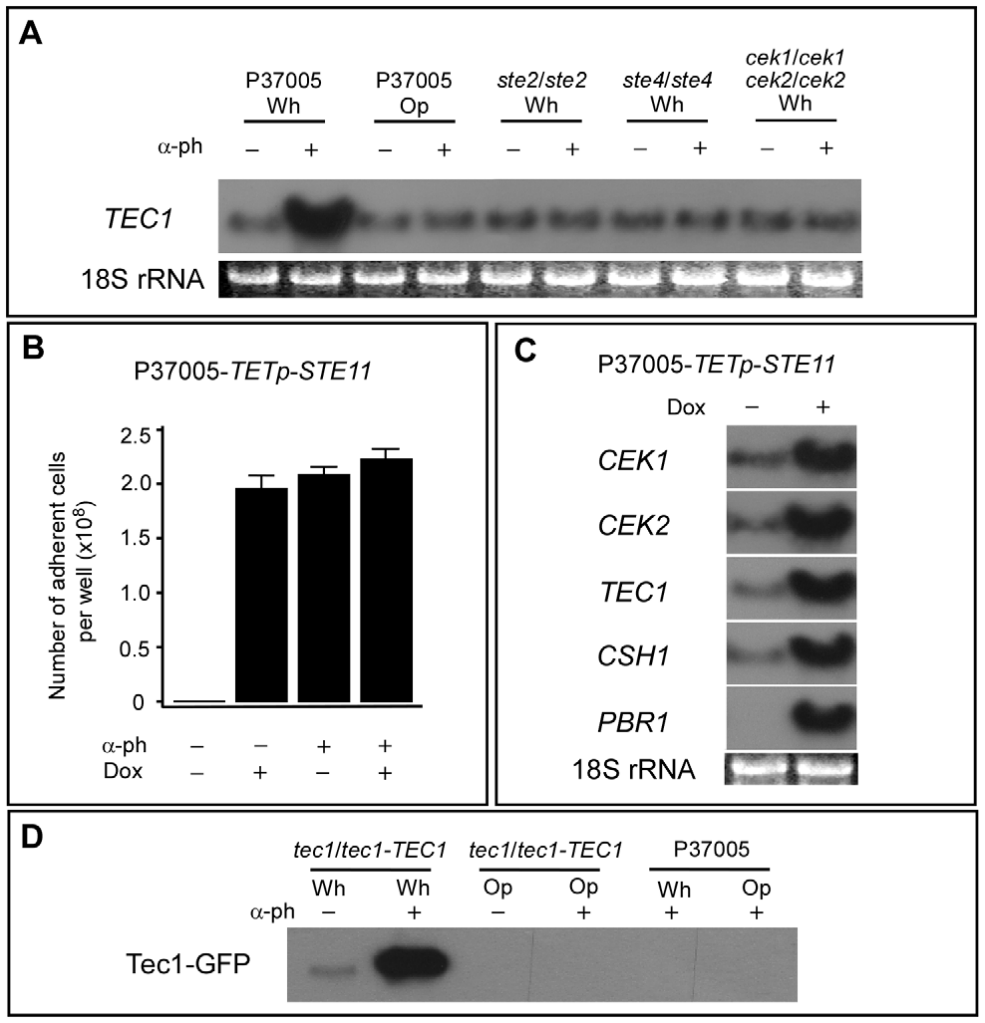
Tec1 functions downstream of the MAP kinase cascade. Up-regulation of *TEC1* by α-pheromone requires the α-pheromone receptor, trimeric G protein complex, and MAP kinase cascade. (A) Northern analysis of pheromone (α-ph) induction of *TEC1* in P37005 white (Wh) and opaque (Op) cells, and in white cells of the mutants *ste2/ste2*, *ste4/ste4*, and *cek1/cek1 cek2/cek2*. (B) Overexpression of *STE11* induces adhesion in the absence of α-pheromone. Overexpression was induced in white cells by adding 50 µg per ml of doxycycline (Dox) to the overexpression strain P37005-*TETp*-*STE11*. (C) Overexpression of *STE11* by addition of 50 µg per ml of doxycycline in the absence of α-pheromone activates genes in the white response pathway in strain P37005-*TETp*-*STE11*. (D) Western blot analysis of Tec1-GFP using anti-GFP antibody in strain *tec1/tec1*-*TEC1* in which *TEC1* is tagged with GFP.

To test whether *TEC1* expression was regulated by α-pheromone and the MAP kinase pathway, a northern analysis was performed to assess its expression in deletion mutants for key elements of the signal transduction pathway in the absence and presence of α-pheromone. In white cells of the control strain P37005, *TEC1* was expressed at a basal level in the absence of α-pheromone, but at a highly elevated level in the presence of α-pheromone (Figure 2A). In opaque cells of P37005, *TEC1* was expressed at the same basal level in the absence and presence of α-pheromone, demonstrating that unlike white cells, α-pheromone did not up-regulate *TEC1* transcription in opaque cells (Figure 2A). In white cells of the deletion mutants of *STE2*, which encodes the α-pheromone receptor, and *STE4*, which encodes the beta subunit of the heterotrimeric G protein complex, and in white cells of the double mutant for *CEK1* and *CEK2*, which encode the two MAP kinase isoforms (*ste2/ste2*, *ste4/ste4*, *cek1/cek1 cek2/cek2*, respectively), α-pheromone did not up-regulate *TEC1* (Figure 2A). Hyperactivating the MAP kinase pathway by overexpressing *STE11*, which encodes the first component of the MAP kinase cascade, in white cells of strain P37005-*TETp*-*STE11* in the absence of α-pheromone, caused an increase in adhesion of over 100-fold (Figure 2B) and up-regulation of *TEC1* transcription (Figure 2C). Overexpression of *STE11* in the absence of α-pheromone also up-regulated *CEK1*, *CEK2*, *CSH1*, and *PBR1* (Figure 2C), genes previously shown to be up-regulated by α-pheromone through the MAP kinase pathway [10],[23]. Overexpression of *STE11* in white cells did not up-regulate opaque-specific genes (unpublished data).

To demonstrate that the protein product of *TEC1*, in addition to the *TEC1* transcript, was selectively up-regulated by α-pheromone in white but not opaque cells, we analyzed the level of Tec1-GFP in strain *tec1/tec1*-*TEC1* in which *TEC1*-GFP had been inserted into its endogenous site under the control of its native promoter (Table S6). The level of Tec1 was assessed by Western blot analysis, using anti-GFP antibody. In the absence of α-pheromone, the basal level of Tec1 in white cells of strain *tec1/tec1*-*TEC1* was extremely low, whereas in the presence of α-pheromone, it was close to two orders of magnitude higher (Figure 2D). In the absence and presence of α-pheromone in opaque cells, the level of Tec1-GFP was undetectable (Figure 2D). Together, these results demonstrate that *TEC1* is selectively up-regulated by α-pheromone at the RNA and protein levels through the MAP kinase pathway in white cells, but not in opaque cells.

In *S. cerevisiae*, Ste12, the homolog of Cph1 in *C. albicans*, regulates *TEC1* expression in the filamentation pathway [32], but in *C. albicans*, Cph2 regulates *TEC1* expression [41]. In the white pheromone response, neither *CPH1* nor *CPH2* are up-regulated by pheromone (unpublished data not shown), whereas *TEC1* is highly up-regulated (Figure 2A), suggesting that neither Cph1 nor Cph2 play a role in *TEC1* regulation in the white pheromone response pathway. Overexpression of *CPH1* in the absence of pheromone did not result in the up-regulation of *TEC1* (Figure 1A).

### Tec1 Regulation of Adhesion and Downstream Genes

**Figure 3:**
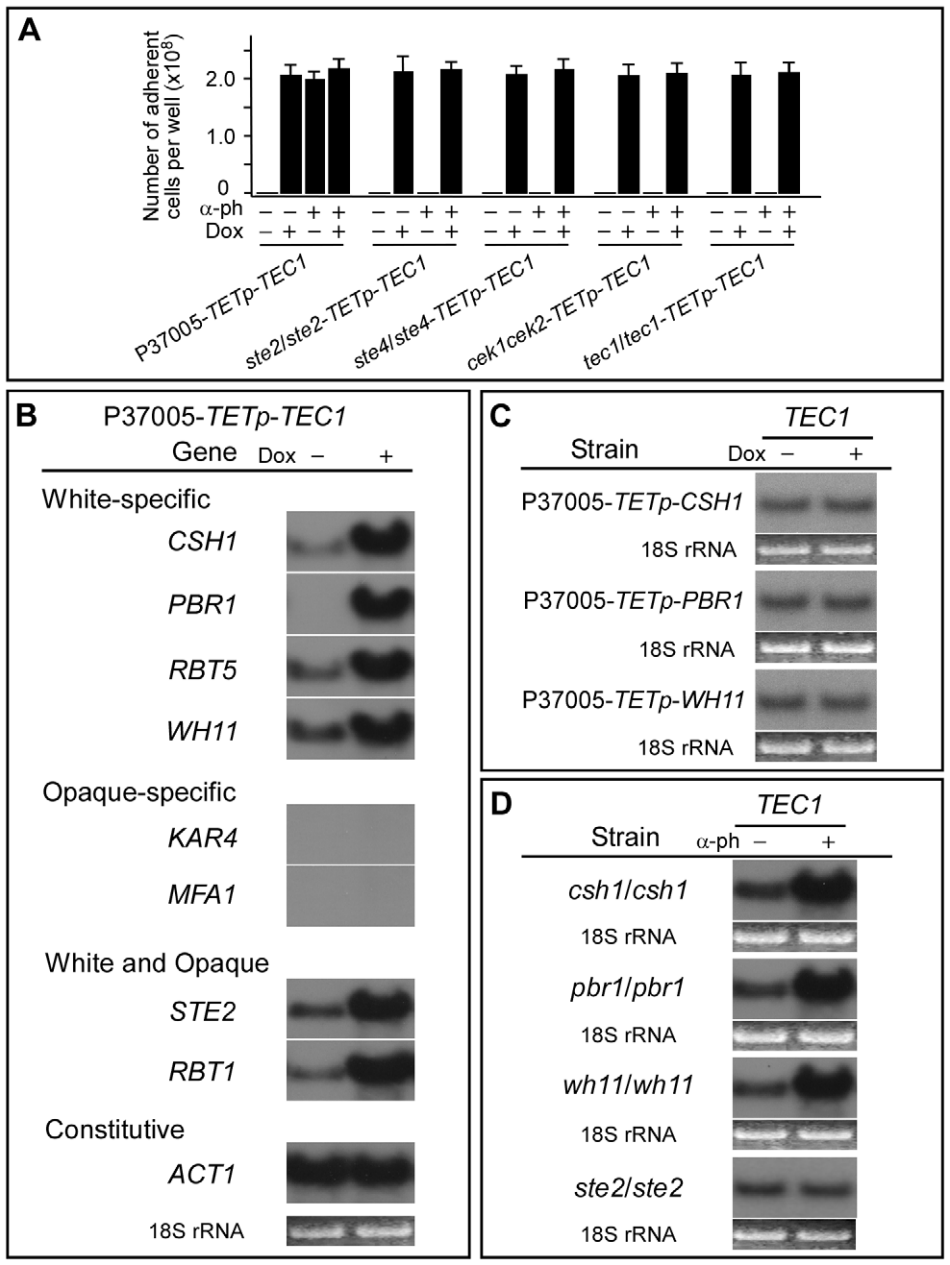
Tec1 regulates pheromone-induced biofilm genes. (A) Adhesion by white cells of *TEC1* overexpressors generated in P37005, *ste2/ste2*, *ste4/ste4*, *cek1/cek1 cek2/cek2*, and *tec1/tec1* in different combinations of α-pheromone (α-ph) and doxycycline (Dox). (B) Northern analysis of gene expression upon overexpression of *TEC1* in strain P37005-*TETp*-*TEC1*. (C) Northern analysis of *TEC1* expression when the target genes *CSH1*, *PBR1*, or *WH11* are overexpressed in strains P37005-*TETp*-*CSH1*, P37005-*TETp*-*PBR1*, and P37005-*TETp*-*WH11*, respectively. (D) Northern analysis of *TEC1* expression in the target gene deletion mutants *csh1/csh1*, *pbr1/pbr1*, and *wh11/wh11* and the receptor deletion mutant *ste2/ste2*.

To demonstrate that Tec1 regulated the white cell pheromone response by up-regulating genes that had been shown to be induced by pheromone in white cells, we tested the effects of *TEC1* overexpression in the absence of α-pheromone in strain P37005-*TETp-TEC1* (Table S3). When *TEC1* was overexpressed in white cells in the absence of α-pheromone, there was an increase in adhesion equivalent to that induced by α-pheromone (Figure 3A). The white-specific genes *CSH1*, *PBR1*, *RBT5*, and *WH11* [10],[23], but not the opaque-specific genes *KAR4* and *MFA1*, were also up-regulated [10],[21],[23],[42] (Figure 3B). The genes *STE2* and *RBT1*, which are up-regulated by α-pheromone in both white and opaque cells [17],[23], were also up-regulated when *TEC1* was overexpressed in white cells in the absence of α-pheromone (Figure 3B). When *TEC1* was overexpressed in the absence of α-pheromone in the mutants *ste2/ste2*-*TETp-TEC1*, *ste4/ste4*-*TETp-TEC1*, and *cek1/cek1 cek2/cek2*-*TETp-TEC1*, adhesion increased as it did in control cells in response to α-pheromone (Figure 3A). These results demonstrate that Tec1 mediates the induction of gene expression by α-pheromone in white cells, and does so downstream of the heterotrimeric G protein complex and MAP kinase cascade.

To test whether downstream genes that were not involved in the signal transduction pathway, but were regulated by Tec1 in turn were involved in up-regulating *TEC1*, we analyzed *TEC1* expression in the strains P37005-*TETp-CSH1*, P37005-*TETp-PBR1*, and P37005-*TETp-WH11*. When these downstream genes were overexpressed in the absence of α-pheromone, the *TEC1* transcript remained at a basal level (Figure 3C). In addition, when *CSH1*, *PBR1*, and *WH11* were deleted, *TEC1* expression was still up-regulated by α-pheromone (Figure 3D). Together, these results demonstrate that Tec1 functions downstream of the MAP kinase pathway, but upstream of the target genes regulated by α-pheromone, and that Tec1-regulated genes, which are not components of the signal transduction pathway, play no role in α-pheromone–induced regulation of *TEC1* expression.

### Tec1 Binds Target Gene Promoters

**Figure 4:**
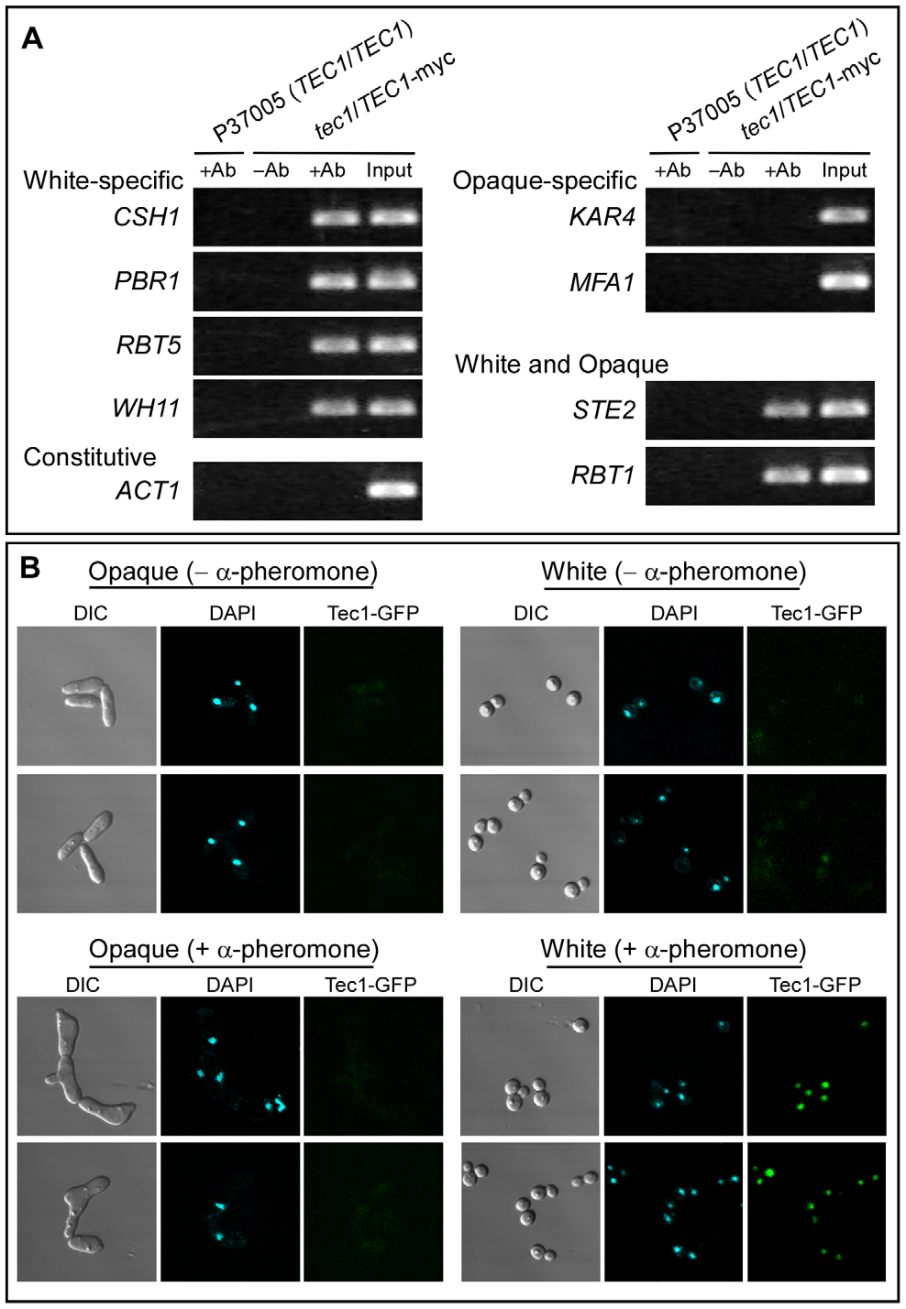
Tec1 directly interacts with WPRE-containing promoter regions and localizes in the nucleus. (A) ChIP analysis of gene promoters that bind to Tec1. The gene categories for the promoters screened for are presented. The primers used for the promoter regions are listed in Table S6. An anti-myc antibody was used to immunoprecipitate chromatin. “Input” represents chromatin preparation before immunoprecipitation, and “−Ab” and “+Ab” represent the absence and presence, respectively, of anti-myc antibody. (B) Opaque and white cells of strain *tec1/tec1*-*TEC1*, in which *TEC1* is tagged with GFP, stained for DNA using DAPI, and imaged for DAPI staining and GFP fluorescence.

Since the transcription factor Tec1 mediates α-pheromone–induced expression of genes in white cells, and WPRE is the presumed *cis*-acting regulatory sequence [23] that mediates up-regulation, we tested whether there was a direct interaction between Tec1 and the promoters of these select genes using a chromatin immunoprecipitation (ChIP) assay [43]–[45]. *TEC1* was tagged with myc at the 3′ end in the heterozygous deletion mutant to generate the strain *tec1/TEC1*-*myc*. White cells of this strain were treated with α-pheromone to induce the putative interaction between Tec1-myc and the promoters of regulated genes. An antibody against myc was then used to immunoprecipitate chromatin fragments bound to Tec1-myc. Immunoprecipitated DNA was then amplified by the polymerase chain reaction with primers designed to span approximately 400 base pairs of the promoter region harboring the WPRE in the case of white-specific genes and the OPRE in the case of opaque-specific genes (Table S6). The white-specific gene promoters tested for were *CSH1*, *PBR1*, *RBT5*, and *WH11*, and the opaque-specific gene promoters tested for were *KAR4* and *MFA1* [10],[17],[23],[42]. We also tested for the promoters of *STE2* and *RBT1*, which contain both a WPRE and OPRE [23]. Finally, we tested for the promoter of *ACT1*, which contains neither a WPRE nor an OPRE [23]. The promoters coimmunoprecipitated by the anti-myc antibody were those of genes selectively up-regulated by α-pheromone in white but not opaque cells (*CSH1*, *PBR1*, *RBT5*, and *WH11*), and those up-regulated by α-pheromone in both white and opaque cells (*STE2*, *RBT1*) (Figure 4A). The promoters of genes up-regulated through Cph1 in opaque cells only (*KAR4*, *MFA1*) and the promoter of the gene *ACT1* were not coimmunoprecipitated by the anti-myc antibody (Figure 4A). These results demonstrate that Tec1 binds selectively to WPRE-containing promoters of the genes up-regulated by α-pheromone in white cells, but not to OPRE-containing promoters of genes up-regulated by α-pheromone only in opaque cells.

### Tec1 Localizes to White Cell Nuclei

Since Tec1 functions as a white-specific transcription factor, is expressed selectively in white cells, and binds to the promoters of genes up-regulated by α-pheromone in white cells, it would be expected to localize to the nuclei of white cells. To test this, we compared DAPI staining, which is specific for DNA and GFP fluorescence, in a strain *tec1/tec1*-*TEC1* that expressed GFP-tagged Tec1 (Table S3). In the absence or presence of α-pheromone, opaque cells of this strain did not exhibit GFP fluorescence either in the cytoplasm or nucleus (Figure 4B). White cells of this strain, however, did exhibit weak GFP fluorescence in the absence of α-pheromone and very intense nuclear fluorescence in the presence of α-pheromone (Figure 4B). The relative GFP fluorescence in the nucleus in the absence and presence of α-pheromone (Figure 4B) reflected the basal and induced levels, respectively, of protein assessed in Western blots (Figure 2D). These results demonstrate that, as expected, Tec1 localizes to the nucleus of white cells.

### Deletion of *TEC1* Abolishes the White Cell but Not Opaque Cell Pheromone Response

**Figure 5:**
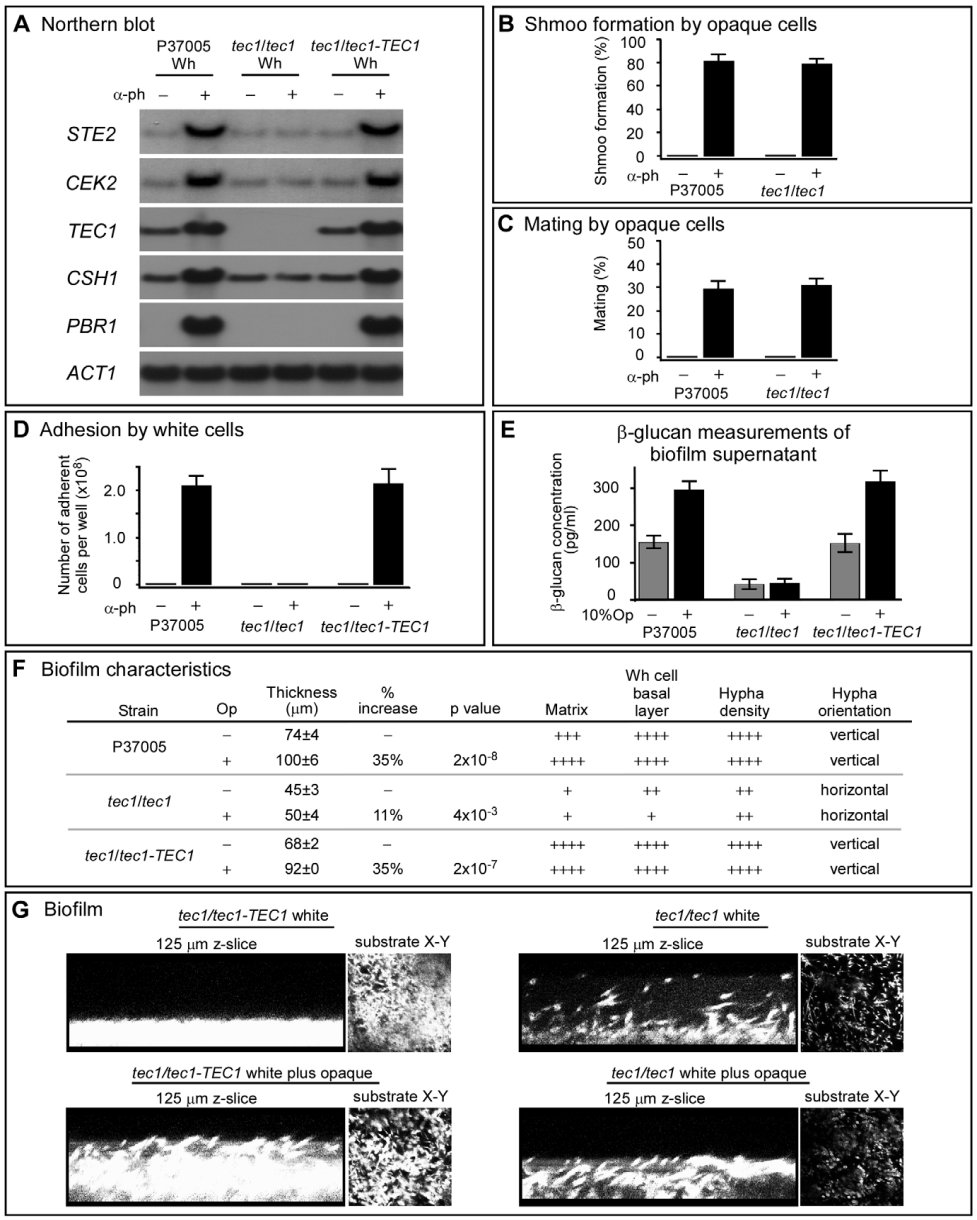
Deletion of *TEC1* results in the loss of the white cell pheromone response. (A) Northern analysis of pheromone-induced gene expression in P37005, *tec1/tec1*, and the complemented strain *tec1/tec1*-*TEC1*. α-ph, α-pheromone; wh, white. (B) Shmoo formation in opaque cells. (C) Mating between opaque cells of P37005 and *tec1/tec1*, which are **a**/**a**, and opaque cells of strain WO-1, which is α/α. (D) Adhesion of white cells on a plastic well bottom. (E) β-Glucan concentration of the supernatant of majority white cell (90%) biofilms in the absence and presence of minority (10%) opaque cells (10%Op) composed of an equal mixture (50∶50) of **a**/**a** and α/α opaque cells. (F) Characterization of *tec1/tec1* biofilms. (G) Side and substrate view of white cell biofilms formed by complemented control cells (*tec1/tec1*-*TEC1*) or deletion mutant cells (*tec1/tec1*).

Deletion of the two copies of *TEC1*, generating the mutant *tec1/tec1* (Table S3), abolished in white cells α-pheromone–induced increases in the transcription of *STE2*, *CEK2*, *CSH1*, and *PBR1* (Figure 5A). If *TEC1* solely mediated the white, but not the opaque, pheromone response, then deletion of *TEC1* should selectively abolish the white cell responses, but should have no effect on opaque cell response to pheromone. This is exactly what was observed. Opaque cells of the *tec1/tec1* mutant formed the same proportion of shmoos in response to α-pheromone as control cells (Figure 5B) and mated with α/α opaque cells at the same frequency as control cells (Figure 5C). In marked contrast, α-pheromone–induced adhesion was abolished in white *tec1/tec1* cells (Figure 5D). In addition, biofilm formation was defective. Thickness in the absence of minority opaque cells was depressed by 39% and 34%, respectively, when compared to wild-type and complemented strains, respectively, and opaque cells stimulated thickness by only 11% in mutant cells versus 35% in cells of both control strains (Figure 5F). The formation of a basal layer of cells was diminished in *tec1/tec1* cells, the orientation of hyphae was horizontal rather than vertical, the latter suggesting a decrease in matrix (Figure 5F). The release of β-glucan into the supernatant, a measure of biofilm matrix formation [46], was diminished 4-fold in *tec1/tec1* cells in the absence of α-pheromone and 8-fold in the presence of α-pheromone, when compared to control cells (Figure 5E). The optical density of the matrix was also noticeably diminished (Figure 5F). The differences in the basal layer, thickness, and hyphae are evident in reconstructed side views and substrate views of confocal images (Figure 5G). These results support the conclusion that *TEC1* is essential both for the formation of a normal white cell biofilm in the absence of minority opaque cells and for the enhancement of white cell biofilm formation in the presence of minority opaque cells.

Interestingly, Bcr1, which has been implicated in the development of hypha-containing biofilms in **a**/α cells [36], is not up-regulated by pheromone in white cells (unpublished data), contains no WPRE in its promoter, and when overexpressed in the absence of pheromone in white cells, does not induce the white cell pheromone response (Figure 1A), indicating it plays no role in the early white cell biofilm response to pheromone.

### Generality of Tec1 Function

Because **a**-pheromone cannot be chemically synthesized due to complex posttranslational modifications [47], studies of the role of pheromone in both *S. cerevisiae* and *C. albicans* have been performed primarily with **a**/**a** strains treated with α-pheromone, which is readily synthesized chemically [19],[48],[49]. To test whether Tec1 played the same role in white α/α cells as it did in white **a**/**a** cells, we generated Tec1 overexpression strains in three natural α/α strains, WO-1 [50], 19F [51], and P57072 [52]. The generated strains were WO-1-*TETp-TEC1*, 19F-*TETp-TEC1*, and P57072-*TETp-TEC1* (Table S3). Adherence was then compared between white cells in the absence and presence of doxycycline. To assess the **a**-pheromone response, we tested adherence in the presence of a 1% mixture of opaque **a**/**a** cells and opaque α/α cells (50∶50), which has been shown to be a source of natural **a**-pheromone under these conditions [17]. For each of the three strains tested, there was over a 100-fold increase in adhesion in doxycycline-treated white cells, approximately the same increase observed for white cells treated with minority opaque cells (Figure S2A). These results indicate that Tec1 mediates the **a**-pheromone response in white α/α cells, as it does in the α-pheromone response in white **a**/**a** cells.

To demonstrate that Tec1 mediates the white cell pheromone response in **a**/**a** strains other than P37005, we tested overexpression of *TEC1* in the absence of α-pheromone in two additional **a**/**a** strains, L26 [53] and P60002 [54], generating strains L26-*TETp-TEC1* and P60002-*TETp-TEC1*. As it did in white cells of the **a**/**a** strain P37005-*TETp-TEC1*, overexpression of *TEC1* in the absence of α-pheromone in strains L26-*TETp-TEC1* and P60002-*TETp-TEC1* resulted in an increase in adhesion similar to that induced by α-pheromone in the absence of doxycycline (Figure S2B), demonstrating the generality of the role of Tec1 in mediating the α-pheromone response among **a**/**a** strains.

## Discussion

The estimated age of *C. albicans* is approximately three million years [55], close to that of the human genus [56]. Here, we have completed the description of what would appear to be a unique and evolutionarily new signal transduction pathway, the *C. albicans* white cell pheromone response pathway. We previously had identified the upstream portion of the pathway from signal receptor through the trimeric G protein complex and MAP kinase cascade [10], and the downstream target genes, including the *cis*-acting consensus sequences in the promoters of those genes that were involved in pheromone activation [23]. Here, we bridged these two portions of the pathway by identifying the transcription factor, targeted by the MAP kinase cascade, that up-regulates pheromone-induced genes in white cells by binding to the white-specific *cis*-acting sequence WPRE. The completed description of this pathway now provides us with unique insights into how new signal transduction pathways may evolve.

### The Key Transcription Regulator

In *S. cerevisiae*, Tec1, a member of the ATTS/TEA family of transcription factors, plays a role in the formation of pseudohyphae [31]–[33]. Tec1 regulates filamentation by binding in a complex that includes Ste12 and the two Ste12 inhibitors Dig1 and Dig2. The complex then binds to the TCS binding motif in the promoters of filamentation genes [35]. In *C. albicans*, Tec1 has also been implicated in enhancing filamentation, but Tec1 is not essential either in vitro or in vivo [34]. Here, we have presented data that Tec1 is the sole downstream target of the MAP kinase cascade. It is up-regulated in white but not opaque cells, mediates the white cell pheromone response, and is essential for the white cell pheromone response. Since the ortholog of Ste12, Cph1, is not up-regulated in the white response, it seems unlikely that Tec1 functions in a complex similar to that regulating filamentation in *S. cerevisiae*.

### Tec1 Functions Through WPRE

We recently demonstrated that genes are up-regulated by pheromone in white cells through the *cis*-acting motif WPRE, which includes the AT-rich consensus sequence AAAAAAAAAAGAAAG [23]. This sequence differs markedly from the *cis*-acting sequence OPRE, which regulates pheromone-inducible genes in the opaque cell pheromone response and includes the GC-rich consensus motif GTGAGGGGA [23]. WPRE contains the six-base motif AGAAAG, which is remarkably similar to the *cis*-acting TCS motif AGAATG [31],[37] that mediates Tec1 binding in *S. cerevisiae*. To demonstrate that Tec1 interacted directly with WPRE-containing promoters of genes up-regulated by pheromone in white cells, we performed ChIP experiments followed by PCR, amplifying a region in each white- or opaque-specific gene promoter that spanned either the WPRE or OPRE, respectively. The results indicated direct interactions. Whereas Tec1 bound to the promoter regions of *CSH1*, *PBR1*, *RBT5*, *WH11*, *STE2*, and *RBT1*, which all contained a WPRE and were all up-regulated by pheromone in white cells, Tec1 did not bind to the promoter regions of *KAR4* or *MFA1*, which contained an OPRE and lacked a WPRE, and did not bind to the promoter of *ACT1*, which lacked both an OPRE and a WPRE [23]. The apparent homology between WPRE in *C. albicans* and TCS in *S. cerevisiae* is noteworthy given the high speed at which transcription factor binding sites have been found to diverge normally in the evolution of yeast [57].

### Tec1 Regulates Biofilm Genes

Tec1 activates both genes that are involved in the signal transduction pathway shared with the opaque cell pheromone response, and genes involved in a variety of membrane or cell wall–associated processes, including adhesion and biofilm formation [23]. In a Northern analysis of 107 genes implicated in adhesion, cell wall biogenesis, biofilm formation, filamentation, and switching, nine genes were found to be strongly up-regulated by pheromone in white but not opaque cells [23]. All of the promoters of these genes as well as those of three additional genes that had been shown to be similarly up-regulated in white cells by pheromone [10],[23], contained a WPRE and lacked an OPRE. Four of these genes randomly selected and deleted were found to play fundamental roles in white cell biofilm formation, suggesting that all or a majority of genes containing a WPRE, but not an OPRE, play a role in α-pheromone–induced white cell biofilm formation. It is noteworthy that all of the biofilm genes up-regulated by Tec1 in the white response in *C. albicans* contain WPRE, and that these same genes (Table S7) in the closely related species *C. dubliniensis* also contain WPRE (Table S8). However, these same genes (Table S7) in *C. tropicalis*, another species of the *Candida* group in the Hemiascomycetes, that does not undergo white-opaque switching, do not contain a WPRE (Table S8).

Interestingly, we found Bcr1, which functions downstream of Tec1 in biofilm formation by **a**/α cells [36], did not play a role in early white cell biofilm formation in response to pheromone. *BCR1* did not contain a WPRE, and was not up-regulated by pheromone in white cells (unpublished data). Overexpression of *BCR1* had no effect on the white cell biofilm response to pheromone (Figure 1A). Mitchell and coworkers [36] specifically discuss this gene as it relates to hypha formation, a much later process in biofilm formation, which may explain why we found no function in early biofilm development. Moreover, it must be kept in mind that there may be fundamental differences between **a**/α biofilms and the *MTL*-homozygous biofilms found in the white response.

### The Evolution of the White Phase Pheromone Response Pathway

**Figure 6:**
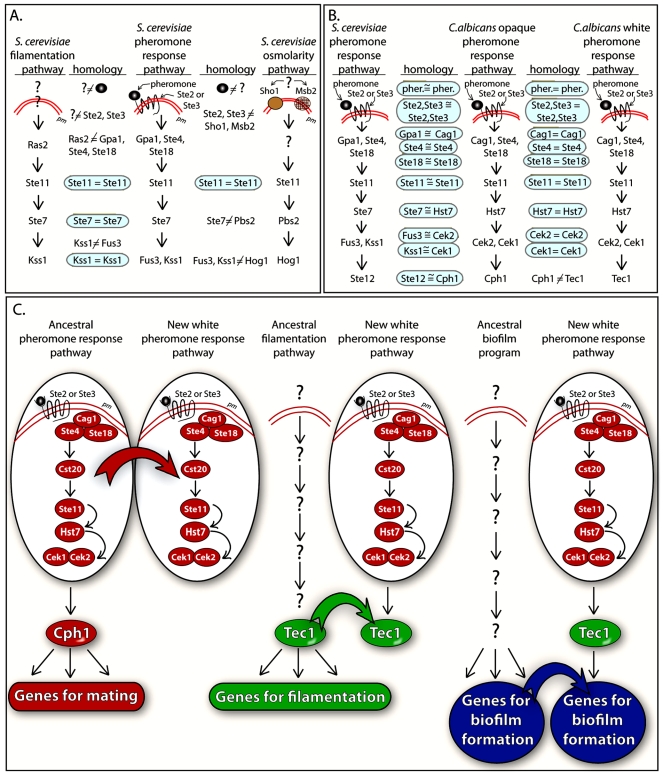
Evolution of the white cell pheromone response pathway. (A) A comparison of common components in the *S. cerevisiae* filamentation pathway, pheromone response pathway, and osmolarity pathway. pm, plasma membrane. (B) A comparison of common components of the *S. cerevisiae* pheromone response pathway, the *C. albicans* opaque cell pheromone response pathway, and the *C. albicans* white cell pheromone response pathway. pher., pheromone. (C) Derivation of the three major portions of the white cell pheromone response pathway.

In *S. cerevisiae*, MAP kinase signaling pathways have been compared for common components between the best understood one, the pheromone response pathway, and a number of others, including those for filamentation, high osmolarity, cell wall integrity, and cell wall assembly [58]. Remarkably, except for the mating pathway, the signal–receptor interactions for the rest are poorly understood. The sharing of common components between these pathways is limited, except for the pheromone response pathway and the filamentation pathway. For these pathways, the GTPase Cdc42 and the kinase Ste20 immediately upstream of the MAP kinase pathway, as with the downstream components of the MAP kinase pathway, Ste11, Ste7, and Kss1, are shared (Figure 6A).

For the white and opaque response pathways of *C. albicans*, a different set of relationships exist. The *C. albicans* opaque cell pheromone response pathway shares with the *S. cerevisiae* haploid cell pheromone response pathway components from the signal to the transcription activator (Figure 6B). The white cell pheromone response pathway, however, shares with the opaque cell pheromone response pathway every component from signal and receptor through the MAP kinase cascade, but targets a different transcription factor, and, hence, a different combination of genes that includes biofilm genes (Figure 6B). We believe that this set of relationships provides unique insights into how signal transduction pathways may evolve.

First, we argue that the white response pathway must be relatively new since white-opaque switching is unique to *C. albicans* and the closely related species *Candida dubliniensis* [16], and probably had evolved in their immediate common ancestor approximately 20 million years ago [55]. White-opaque switching has not been observed in any of the other members of the Hemiascomycetes. We propose that in the evolution of the white pheromone response pathway, all of the components were derived from components in other ancestral pathways that had been conserved and still serve the same functions in *C. albicans*. First, we propose that the components in the upstream portion of the white pathway, including signals, receptors, the coupled trimeric G protein complex, and the MAP kinase cascade, were borrowed intact from the ancestral mating pathway (step **a** in Figure 6C), as evidenced by the strict homology between components of the white cell pheromone response pathway, the opaque cell pheromone response pathway, and the *S. cerevisiae* haploid cell pheromone response pathway (Figure 6B) [10],[21]. Second, we argue that the transcription factor regulated by the MAP kinase cascade in the white cell pheromone response pathway, Tec1, was borrowed from an ancestral filamentation pathway (step b in Figure 6C), as is suggested by its conserved role in *S. cerevisiae* filamentation [32],[35] and *C. albicans* filamentation [34]. We argue that Tec1 was not borrowed in a single step with downstream target genes involved in biofilm formation, because those genes that possess a Tec1 binding site (WPRE) in *C. albicans*, do not possess that site in *C. tropicalis*, suggesting that Tec1 may not have regulated biofilm genes in a common ancestor. Finally, we propose that the genes directly regulated by Tec1 and involved in the formation of white cell biofilms were borrowed from an ancestral system for biofilm formation, which remains active in biofilm formation in **a**/α cells of *C. albicans* (step c in Figure 6C). The *cis*-acting WPRE element evolved in the promoters of these genes for responsiveness to Tec1. The steps in Figure 6C reflect what we believe to be three discrete evolutionary events and are not supposed to reflect any temporal order.

The signal transduction pathway of the white cell pheromone response evolved to facilitate the outcome of the pathway from which it was derived, namely the mating process. Hence, the upstream portions of the original (opaque) and derived (white) pathways share the same signal and receptor for coordination (Figure 6C). However, since the specific outcomes of the original and derived pathways differ (i.e., mating versus biofilm development), the derived white cell pathway borrowed target genes from another pathway with the necessary phenotypic outcome, namely the ancestral biofilm program, still functioning in **a**/α strains (Figure 6C) [59]–[61]. To glue the upstream and downstream components together, the transcription factor Tec1 was borrowed from a third developmental program, filamentation (Figure 6C). We therefore suggest that all of the components of the white cell pheromone response pathway have been borrowed from ancestral programs that are still relatively intact and functional. The white pathway is young and thus has had insufficient time to duplicate, replace, or modify components in response to new selective pressures or changing roles. We suggest that the evolution of the white cell pheromone response pathway in *C. albicans* affords a unique glimpse into the evolution of a new signal transduction pathway, and may provide at least one paradigm for how such pathways may evolve.

## Materials and Methods

### Yeast Strains and Growth Conditions

The yeast strains used in this study and their genotypes are listed in Table S3. Cells of all natural strains and the derived mutants were maintained at 25°C on agar plates containing modified Lee's medium [62],[63] supplemented with 5 µg/ml phloxine B, which differentially stained opaque colonies and sectors red [64]. Cells in the white or opaque phase were also verified microscopically prior to use.

### Construction of a Transcription Factor Overexpression Library

A total of 107 genes encoding putative transcription factors in *C. albicans* were selected based on their function in cell wall/membrane biogenesis, basic metabolism, adhesion, filamentation, or biofilm formation. The functions were derived by Gene Ontology (GO) term searches in the *Candida* genome database (http://www.candidagenome.org/). The annotations of these transcription factors are described in Table S1.

The plasmid pNIM1 harboring a *GFP* gene and a tetracycline-inducible promoter *TETp* [30] was used to generate the overexpression module for each transcription factor gene. This plasmid was a generous gift from Joachim Morschhäuser from the University of Würzburg, Germany. The ORFs of the 107 genes encoding transcription factors were amplified by PCR using the primer sets listed in Table S2. Each of the PCR products was digested with SalI or XhoI, and cloned into the SalI-cut, dephosphorylated plasmid pNIM1. The derived plasmids were verified by sequencing for fusion of the GFP ORF in-frame to the 3′ of each gene. The plasmids were then linearized by ApaI or SacII enzyme digestion, and transformed into the **a**/**a** natural *C. albicans* strain P37005 to generate a library (Table S3). The library strains were confirmed by PCR.

### Adhesion Assay

α-Pheromone–induced adhesion was assessed following incubation of white cells of **a**/**a** strains at a concentration of 5×10^7^/ml in fresh modified Lee's medium at 25°C in the wells of a Costar 12-well cluster plate (Corning Life Sciences) in the presence or absence of 3×10^−6^ M synthetic 13-mer α-pheromone with a sequence of GFRLTNFGYFEPG (Open Biosystems) according to the methods previously discussed [10],[17]. The synthetic α-pheromone was dissolved in dimethyl sulfoxide (DMSO). For controls in the absence of pheromone, an equivalent amount of DMSO was added. After 16 h of incubation, the wells were washed gently with phosphate-buffered saline, and the bottoms of the wells were imaged. Adhesion was then quantified by releasing cells from the dish bottoms using 0.05% trypsin-EDTA solution (Invitrogen), and counting the total number of adherent cells in a hemocytometer. For comparative purposes, the means and standard deviations of three independent wells for each strain were calculated and presented in a bar chart. The adhesion experiment and all other experiments in this study were repeated at least twice, and similar results were obtained.

### Motif Elicitation by MEME Analysis

To identify common *cis*-acting DNA motifs, genes regulated in *C. albicans* by Cph1 or Tec1 and genes regulated in *S. cerevisiae* by Ste12 or Tec1 were analyzed (see Table S4). One thousand base pairs upstream of the translation start codon of the ORFs of each set of 17 genes (Table S4) were subjected to MEME analysis to identify a putative consensus sequence (http://meme.sdsc.edu/meme/cgi-bin/meme.cgi). A stringency threshold E value less than 10^−3^ was used as previous described [23],[38]–[40]. The program output with the lowest E value for each gene set represented the best putative consensus DNA motif. Logos were prepared using Weblogo (http://weblogo.berkeley.edu/logo.cgi). For comparative analysis, the promoters of *C. dubliniensis* and *C. tropicalis* orthologs of genes regulated by Tec1 in *C. albicans* were also employed (see the gene list in Table S7) for MEME analysis, and the optimal consensus DNA motif was generated in each species (Table S8). In each promoter, the position of the *cis*-acting DNA motif with the highest homology to the consensus sequence is presented relative to the translation start codon (Table S8).

### Northern Blot Hybridization

Northern blot hybridization was performed as described previously [10],[45]. Cells from stationary-phase cultures were resuspended in fresh modified Lee's medium and incubated for 4 h in the absence or presence of 3×10^−6^ M α-pheromone or 50 µg/ml of doxycycline, an analog of tetracycline. Cells were then spun down and the total RNA was extracted using the RNeasy Mini kit (Qiagen). RNA was separated on a 1.2% formaldehyde agarose gel, transferred to a Hybond-N+ nylon membrane (Amersham), and hybridized with a ^32^P-labelled probe. The Northern blot membrane was exposed to an autoradiographic Kodak film (Eastman Kodak). Two independent samples were subjected to Northern analysis for each experimental setting, and one representative image was presented in the figures. The primers for synthesizing the Northern probes are described in Table S6.

### Hyperactivation of the MAP Kinase Pathway

Hyperactivation of the MAP kinase pathway was achieved by overexpression of the MAPKKK gene *STE11* in the **a**/**a** strain P37005 (Table S3), using the same strategy as that for construction of the overexpression library. Gene induction by doxycycline was confirmed by Northern blot hybridization and fluorescence microscopy. The primer pairs used for generating the *STE11* ORF are described in Table S6.

### Western Blot

*C. albicans* cells were harvested following 4 h of α-pheromone treatment in liquid modified Lee's medium. Total protein of each sample was extracted, and the protein concentration was quantified in Coomassie Plus protein assay reagent (Pierce) according to the Bradford method described previously [21]. An equal amount of each protein sample was subjected to 8% SDS-polyacrylamide gel electrophoresis (PAGE). The samples were then transferred to a PVDF membrane (Immobilon-P, Millipore Corporation). After blocking, the membrane was incubated first with rabbit anti-GFP polyclonal antibody (SC-8334, Santa Cruz Biotechnology) at 4°C overnight, and then incubated with horseradish peroxidase–labeled goat anti-rabbit IgG (Promega). Finally, the protein signal on the membrane was detected with SuperSignal West Pico Chemiluminescent Substrate (Pierce) and exposed to autoradiographic film (Eastman Kodak). Two replicates were included in the Western blot analysis, and similar results were obtained.

### Generation of Homozygous Deletion Mutants and Complemented Strains

The recyclable cassette SAT1-2A harboring the marker SAT^r^ was used for generating the mutant strains. The plasmid pSFS2A was also a generous gift from Joachim Morschhäuser. To generate the homozygous deletion mutant of *TEC1*, a two-step PCR disruption strategy was used. For the first deletion cassette, the 5′ and 3′ flanking regions of *TEC1* were amplified by PCR using the primer pairs listed in Table S6. The 5′ and 3′ regions were then digested with SmaI and ligated using T4 ligase. The 5′-3′ ligation product was then amplified by PCR and cloned into the pGEM-T Easy vector (Promega), yielding the plasmid pTEC1-T1. SAT1-2A was then ligated into the SmaI-digested, dephosphorylated plasmid pTEC1-T1, yielding pTEC1-T1-2A. This plasmid was digested with SacI plus SacII, and transformed into the wild-type strain P37005 by electroporation [65]. The transformants were confirmed as heterozygous by both PCR and Southern analysis. The heterozygotes were then to grown in YPM medium [10],[21] to excise the SAT^r^ marker. The second deletion cassette was generated similarly with primer pairs listed in Table S6, and was used to transform the heterozygous mutants, deriving homozygotes. The homozygous deletion mutants were verified by both PCR and Southern analysis.

For generating a *TEC1*-complemented strain, the 5′ and 3′ regions flanking the stop codon were amplified by PCR using the primers listed in Table S6. The 5′-3′ fusion product was then amplified by PCR and subcloned into pGEM-T Easy (Promega). The DNA fragment GFP-*CaSAT1* was amplified by PCR from the plasmid pK91.6 [10], digested with BamHI plus BglII, and ligated into the BamHI-cut, dephosphorylated plasmid containing the 5′-3′ ligation product, yielding the plasmid pTEC1-comp. The *TEC1*-GFP in-frame fusion was verified by sequencing. Finally, the plasmid pTEC1-comp was digested with SacI plus SacII, and transformed into *tec1/tec1* to generate *tec1/tec1*-*TEC1*, which was verified by both PCR and Southern analysis.

### Generation of *MYC*-tagged Strains and ChIP-PCR Analysis

To generate a *MYC*-tagged *TEC1* strain, the 5′ and 3′ regions flanking the stop codon of the *TEC1* gene were amplified by PCR, using the primers listed in Table S6. The 5′ and 3′ regions were then digested with XbaI and ligated using T4 ligase. The ligation product was cloned into the pGEM-T Easy vector (Promega), yielding pTEC1-T. A DNA fragment harboring a 13 × Myc epitope tag and a dominant nourseothricin marker SAT^r^ was amplified by PCR using primers listed in Table S5 and the plasmid p13myc-natMX as the template [57]. This fragment was cloned into the XbaI-cut, dephosphorylated plasmid pTEC1-T, yielding pTEC1-myc. This plasmid was verified by sequencing for correct in-frame fusion and the number of Myc units, and by Western blot analysis for expected molecular weight and protein expression levels. The plasmid pTEC1-myc was then linearized, digested with SacI and SacII, and transformed into the heterozygous deletion mutant of *TEC1*, generating the *MYC*-tagged *TEC1* strain. This *MYC*-tagged strain behaved similarly to its parental wild-type strain under all experimental conditions.

To test whether the Myc-tagged Tec1 protein complex bound to a specific DNA target, a ChIP-PCR analysis was performed. The detailed description for ChIP-PCR can be found in Protocol S1. The Tec1-binding sites in different species are summarized in Table S5.

### Visualization of GFP-Tagged Proteins

For GFP visualization, white and opaque cells of the *TEC1* complemented strain, which harbored a GFP-tagged Tec1, were grown for 48 h in modified Lee's medium [63]. The cells were pelleted and resuspended in fresh medium, treated with 3×10^−6^ M synthetic α-pheromone for 4 h, and then fixed in 1% formaldehyde in Dulbecco's phosphate buffered saline (Gibco). Nuclei were counterstained for DNA with DAPI. The untagged strain P37005 was included in this analysis as a negative control. Fluorescence was visualized through a Nikon TE2000 microscope attached to a Bio-Rad MP2100 laser scanning confocal microscope equipped with a Mai-Tai infrared laser (Spectra Physics). Sequential images of GFP, DAPI, and transmitted light were acquired. More than 1,000 cells were examined in each sample under all experimental conditions.

### Shmoo and Mating Analyses

The methods for assaying shmoo formation in response to 3×10^−6^ M synthetic α-pheromone, and mating with opaque cells of the α/α strain WO-1 have been described previously in detail [10],[17]. In brief, opaque cells of different strains were grown in liquid modified Lee's medium at 25°C until stationary phase. Cells were then spun down and resuspended in fresh medium at a concentration of 10^7^ cells/ml in the absence or presence of 3×10^−6^ M α-pheromone, and incubated at 25°C for 4 h. Shmoo formation was then monitored microscopically, and the percentage of shmooing cells was quantified from a total of 1,500 randomly selected cells. For mating assay, opaque cells of an **a**/**a** mutant or wild-type strain P37005 were resuspended in fresh liquid medium from a stationary phase culture, and mixed with an equal amount of opaque cells of the α/α strain WO-1. The mating mixture was incubated at 25°C for 24 h before it was monitored microscopically for fusants. Mating efficiency was computed as the percentage of cells that fused out of a total number of 5,000 randomly selected cells. The means and standard deviations for percent shmoo formation and mating efficiency from three independent experiments are presented in bar charts.

### Quantitation of Biofilm Formation

Biofilm enhancement by minority opaque cells was assessed by adding 5% opaque **a**/**a** P37005 cells and 5% opaque α/α WO-1 cells. The cell mixture was incubated at 29°C on silicone elastomer squares (Cardiovascular Instrument Corp.) in RPMI medium in a well of a 12-well cluster plate (Costar, Corning Inc.) for 90 min [10],[17]. The squares were then washed gently with phosphate buffer saline (PBS), placed in a new well with fresh RPMI medium, and further incubated on a rocker (Immunetics) for 48 h. The biofilm was then fixed by addition of 10% formaldehyde to the culture, rinsed with PBS, and stained with calcofluor (Fluorescent Brightener 28, Sigma) in 0.1 M Tris (pH 9.0) and imaged as above. Calcofluor was excited at 818 nm. Using BioRad LaserSharp software, an initial *x-y* optical section was gathered at the biofilm–substrate interface. Thickness of the biofilm in the same field was determined by a 125-µm *Z*-series 2.0-µm steps. Biofilms were prepared in triplicate cultures, and the means and standard deviations of biofilm thickness from three samples were presented. The characteristics of the biofilm (matrix, hyphae, etc.) were determined by scrolling through the *Z*-series. A single *Z-X* slice through the *Z*-series was then digitally acquired.

Biofilm matrix formation was quantitated by measuring the concentration of (1,3)-β-glucan in the biofilm supernatant, according to the methods previously described in detail [23],[46]. Glucan concentration was measured using Glucatell (1,3)-β-d-Glucan Detection Reagent Kit (Associates of Cape Cod) according to the manufacturer's instructions. The glucan concentration was assessed by an end-point assay in which the optical density values were measured at 540 nm in a microplate reader (MDS Analytical Technologies). The means and standard deviations of (1,3)-β-glucan concentration from the three independent biofilm samples are presented in a bar chart.

### Accession Numbers

Detailed information for the genes from *S. cerevisiae* can be found at the *Saccharomyces* Genome Database (http://www.yeastgenome.org/), and information for the genes from *C. albicans* can be found at the *Candida* Genome Database (http://www.candidagenome.org). Information on the *TEC1* homologs in other species can be found at the National Center for Biotechnology Information (NCBI) Web site (http://www.ncbi.nlm.nih.gov/).

## Supporting Information

Figure S1 **Alignment of Tec1 DNA-binding domain across yeast ascomycetes lineages.**
(0.17 MB DOC)

Figure S2 **The role of Tec1 in the white cell response to pheromone is general among natural strains.**
(0.06 MB DOC)

Protocol S1 **ChIP-PCR.**
(0.04 MB DOC)

Table S1 **Overexpression library of *C. albicans* transcription factors.**
(0.14 MB DOC)

Table S2 **Oligonucleotides used for library construction in this study.**
(0.22 MB DOC)

Table S3 ***C. albicans* strains used in this study.**
(0.18 MB DOC)

Table S4 **List of genes used for MEME analysis.**
(0.05 MB DOC)

Table S5 ***cis*-Acting DNA motifs bound by Tec1 homologs in different species.**
(0.05 MB DOC)

Table S6 **Oligonucleotides used for mutant construction, Northern analysis, and ChIP-PCR.**
(0.08 MB DOC)

Table S7 **Promoter comparative analysis using gene orthologs in *C. albicans***, ***C. dubliniensis*, and *C. tropicalis* in MEME.**
(0.05 MB DOC)

Table S8 **Elicitation of *cis*-acting consensus regulatory motifs from the promoters of orthologs of Tec1-target genes in the three fungal species *C. albicans*, *C. dubliniensis*, and *C. tropicalis*.**
(0.10 MB DOC)

## Abbreviations

ChIP: chromatin immunoprecipitation
