# Supplementary material for: Tec1 Mediates the Pheromone Response of the White Phenotype of Candida albicans: Insights into the Evolution of New Signal Transduction Pathways
Source: PLoS Biol. 2010 May 4;8(5):e1000363. doi: 10.1371/journal.pbio.1000363 (PMC2864266; doi:10.1371/journal.pbio.1000363)
Supplement: Figure S1 — Alignment of Tec1 DNA-binding domain across yeast ascomycetes lineages. (0.17 MB DOC) [file pbio.1000363.s001.doc]

**Supporting information**

**Supplemental Figure S1.**


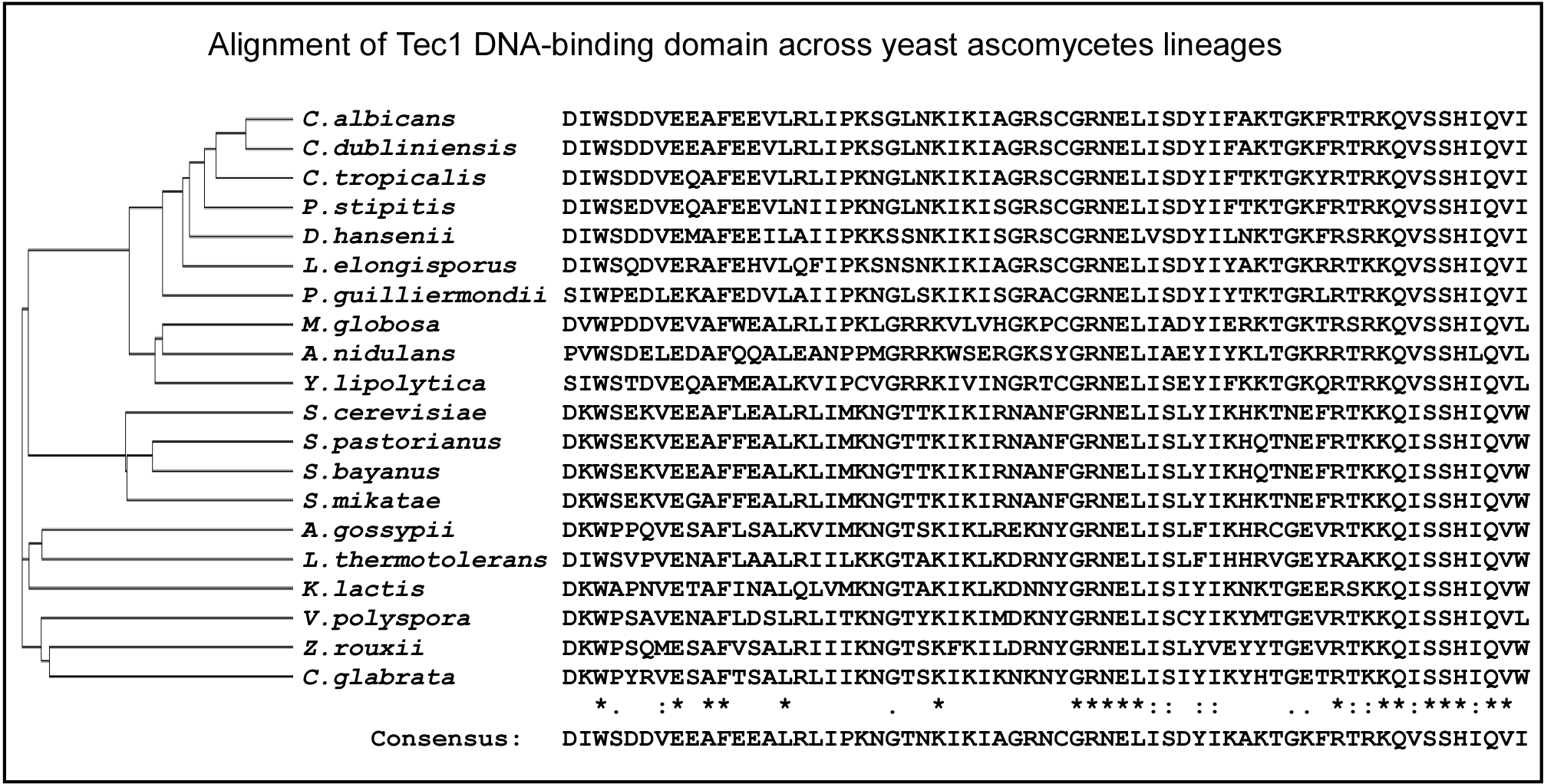


**Supplemental Figure S1 legend**

The amino acid sequences of Tec1 DNA-binding domain were compared among 20 yeast species in the ascomycetes lineage. The sequences were aligned with the Clustal W program [1,2] available at http://www.ebi.ac.uk/clustalw. Identical amino acid residues were denoted by stars ‘*’, while conservative residues were denoted by either ':' (based on similar functional groups) or '.' (based on similar effects on secondary structure). Based on the alignment, a phylogenetic tree for the amino acid sequences in these 20 species was generated. Note, the DNA-binding domain of Tec1 is highly conserved across yeast ascomycetes lineages.

References

1. Higgins D, Thompson J, Gibson T, Thompson JD, Higgins DG, et al. (1994) CLUSTAL W: improving the sensitivity of progressive multiple sequence alignment through sequence weighting, position-specific gap penalties and weight matrix choice. Nucleic Acids Research 22: 4673-4680.
2. Larkin MA, Blackshields G, Brown NP, Chenna R, McGettigan PA, et al. (2007) ClustalW and ClustalX version 2. Bioinformatics 23: 2947-2948.
