# Supplementary material for: Tec1 Mediates the Pheromone Response of the White Phenotype of Candida albicans: Insights into the Evolution of New Signal Transduction Pathways
Source: PLoS Biol. 2010 May 4;8(5):e1000363. doi: 10.1371/journal.pbio.1000363 (PMC2864266; doi:10.1371/journal.pbio.1000363)
Supplement: Figure S2 — The role of Tec1 in the white cell response to pheromone is general among natural strains. (0.06 MB DOC) [file pbio.1000363.s002.doc]

**Supporting information**

**Supplemental Figure S2. The role of Tec1 in the white cell response to pheromone is general among natural strains.**


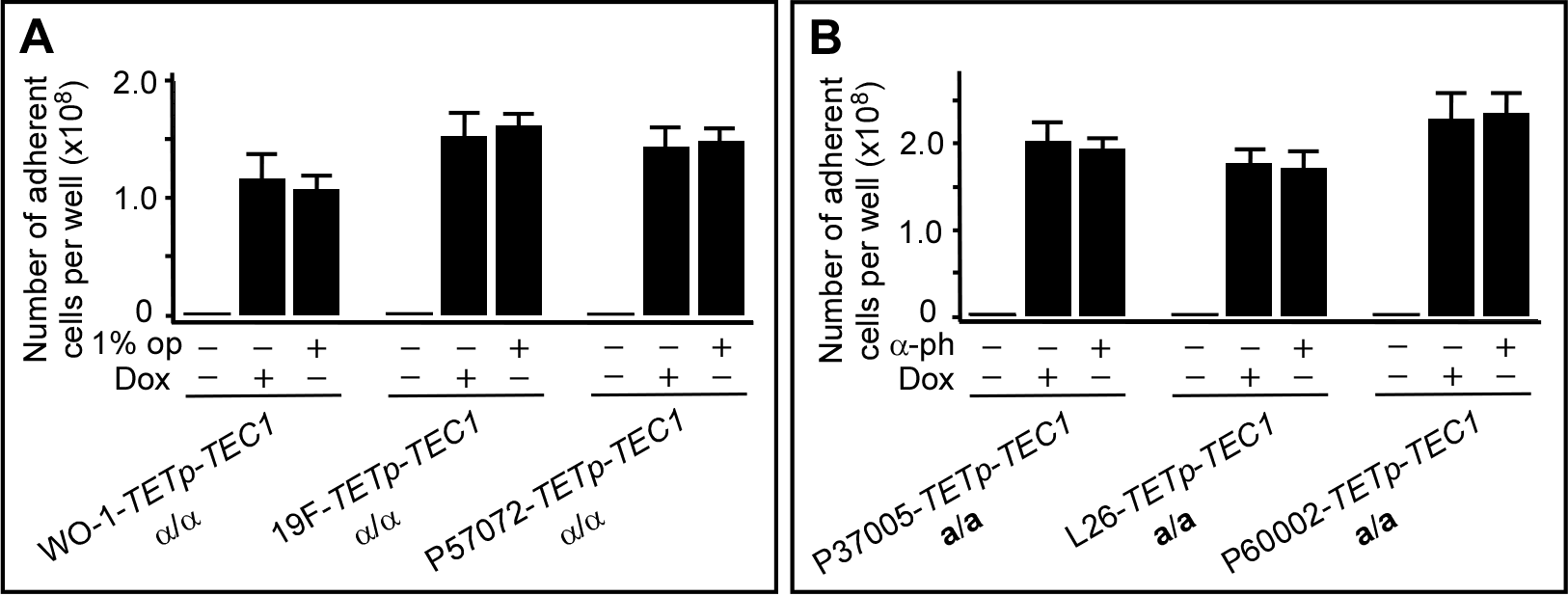


**Supplemental Figure S2 legend**

A. The effects of **a**-pheromone and the overexpression of *TEC1* on adhesion in three natural α/α strains. In this case, pheromone induction was accomplished by adding to a majority (99%) population of white cells a minority (1%) mixture of opaque **a**/**a** and opaque α/α cells (50:50) [1, 2]. B. The effects of α-pheromone and the overexpression of *TEC1* on adhesion in three natural **a**/**a** strains.

References

1. Yi S, Sahni N, Daniels KJ, Pujol C, Srikantha T, et al. (2008) The same receptor, G protein, and mitogen-activated protein kinase pathway activate different downstream regulators in the alternative white and opaque pheromone responses of *Candida albicans*. Mol Biol Cell 19: 957-970.
2. Daniels KJ, Srikantha T, Lockhart SR, Pujol C, Soll DR (2006) Opaque cells signal white cells to form biofilms in *Candida albicans*. EMBO J 25: 2240-2252.
