# Supplementary material for: Tec1 Mediates the Pheromone Response of the White Phenotype of Candida albicans: Insights into the Evolution of New Signal Transduction Pathways
Source: PLoS Biol. 2010 May 4;8(5):e1000363. doi: 10.1371/journal.pbio.1000363 (PMC2864266; doi:10.1371/journal.pbio.1000363)
Supplement: Protocol S1 — ChIP-PCR. (0.04 MB DOC) [file pbio.1000363.s003.doc]

**Supporting information**

**Supplemental Protocol S1. ChIP-PCR**

1. **Chromatin immunoprecipitation (ChIP)**

The myc-tagged Tec1 strain and control strains were grown to saturation phase. Cells were resuspended in fresh liquid Lee’s medium at a concentration of 5×10^7^/ml, and incubated in the presence or absence of α-pheromone at 25^o^C for 4 hr, with a final OD_600_ of 0.6-1.0. The cultures were then fixed to crosslink the protein-DNA complexes in cells by adding formaldehyde at a final concentration of 1%, and incubated in a slow shaker for 15 minutes. Glycine was then added to quench crosslinks at a concentration of 125 mM followed by incubating for five minutes. The cells were harvested by centrifugation at 4°C for 10 minutes at 4000 rpm. The pellets were then washed twice in ice cold 1X TBS (20 mM Tris-HCl, 150 mM NaCl, pH 7.6) prior to lysis. Cell lysis was done by resuspending the pellet in ice-cold lysis buffer (0.1% deoxycholic acid, 1 mM EDTA, 50 mM HEPES/KOH, pH 7.5, 140 mM NaCl, 1% Triton X-100) with protease inhibitors cocktail (Sigma-Aldrich, St. Louis, MO) and 1 mM phenylmethylsulphonyl fluoride (PMSF). Zirconium beads were added to the cell pellets and the cells were lysed by bead beating 7-8 times using Mini Beadbeater-8 (BioSpec Products Inc., Bartlesville, OK), 2 minutes each. Between the bead beatings, the cells were incubated on ice for one minute. The lysate was collected by centrifugation at 4°C for 10 minutes at 4000 rpm.

In the lysate, the chromatin was sheared by sonication in Vibra Cell sonicator (Sonics & Materials Inc., Newtown, CT) (amplitude 20%) 9 times, 10 seconds each. In between sonication pulses, the cells were incubated on ice for one minute. The sheared chromatin averaged 500 to 800 bp in length. 50 µl of extract was stored at -20°C as the ChIP “Input” material. For chromatin IPs, the rest of the cell extract was pre-cleared by incubation with protein G sepharose beads (Active Motif, Carlsbad, CA) for 2 hr at 4°C, and then mixed with anti-c-myc antibody coupled EZview red beads (E6654**,** Sigma-Aldrich, St. Louis, MO) and incubated on a rotating platform overnight at 4°C. The following day, the beads were washed at room temperature first in lysis buffer, next in high salt lysis buffer (0.1% deoxycholic acid, 1 mM EDTA, 50 mM HEPES/KOH, pH 7.5, 500 mM NaCl, 1% Triton X-100) , then in wash buffer (0.5% deoxycholic acid, 1 mM EDTA, 10 mM Tris-HCl, pH 8.0, 250 mM LiCl, 0.5% NP-40, 0.5% sodium deoxycholate) and last in 1X TBS. Finally the immunoprecipitates were eluted by adding 25μl of elution buffer (50 mM Tris/HCl, pH 8.0, 10 mM EDTA, 1% SDS) and incubating at 65°C for 10 min. The beads were spun for 1 minute at 10,000*g* at room temperature. A second elution was done by adding 25μl of elution buffer 2 (50 mM Tris/HCl, pH 8.0, 10 mM EDTA, 0.67% SDS). Both the ChIP and the input samples were incubated overnight at 65°C to reverse the crosslinks. To further remove the proteins, the samples were treated with proteinase K at 37°C for 2h. The DNA was finally purified using the PCR purification kit (QIAGEN, Valencia, CA) and used as PCR template.

1. **PCR assays**

Primers were designed for the promoter region of each tested gene (supplemental Table S6). The immunoprecipitated DNA was amplified by PCR under the following thermalcycling conditions: 95°C for 5 min, 30 cycles of 25 sec at 94°C, 30 sec at 50°C, 25 sec at 72°C, followed by a 7-min extension time at 72°C. Input DNA from sonicated lysate was amplified in parallel. The PCR products were subjected to a 0.8% agarose gel electrophoresis and visualized by ethidium bromide staining.
