# Supplementary material for: Tec1 Mediates the Pheromone Response of the White Phenotype of Candida albicans: Insights into the Evolution of New Signal Transduction Pathways
Source: PLoS Biol. 2010 May 4;8(5):e1000363. doi: 10.1371/journal.pbio.1000363 (PMC2864266; doi:10.1371/journal.pbio.1000363)
Supplement: Table S1 — Overexpression library of C. albicans transcription factors. (0.14 MB DOC) [file pbio.1000363.s004.doc]

**Supporting information**

**Supplemental Table S1. Overexpression library of *C. albicans* transcription factors.**

| No. | Gene/ ORF | Sc. homolog | DNA-binding motif | Function (*Candida* genome database) |
| --- | --- | --- | --- | --- |
|  |  |  |  |  |
| 1 | *ACE2* | *ACE2* | Zinc finger | morphogenesis, adherence, biofilm formation and virulence |
| 2 | *ADA2* | *ADA2* | Zinc finger | cell wall integrity and resistance to caspofungin |
| 3 | *ASH1* | *ASH1* | Zinc finger | filamentous growth and virulence |
| 4 | *BCR1* | *USV1* | Zinc finger | biofilm formation and regulation of cell-surface genes |
| 5 | *BDF1* | *BDF1* | TATA binding | adherence to polystyrene |
| 6 | *BRE1* | *BRE1* | Zinc finger | filamentous growth |
| 7 | *CAP1* | *YAP1* | bZIP | multidrug resistance and oxidative stress response |
| 8 | *CAS5* | *YGL035C* | Zinc finger | cell wall integrity and resistance to caspofungin |
| 9 | *CPH1* | *STE12* | STE-like | filamentous growth and mating |
| 10 | *CRZ1* | *CRZ1* | Zinc finger | multidrug resistance and oxidative stress response |
| 11 | *CRZ2* | *CRZ1* | Zinc finger | multidrug resistance and oxidative stress response |
| 12 | *CSR1* | *ZAP1* | Zinc finger | biofilm matrix formation and filamentous growth |
| 13 | *CTA4* | *OAF1* | Zinc cluster | adherence to polystyrene |
| 14 | *CWT1* | *RDS2* | Zinc finger | cell wall biogenesis |
| 15 | *EFG1* | *SOK2* | bHLH | filamentous growth, adherence and virulence |
| 16 | *EFH1* | *SOK2* | APSES domain | filamentous growth, adherence and virulence |
| 17 | *FCR1* | *CAT8* | Zinc cluster | filamentous growth and fluconazole resistance |
| 18 | *FCR3* | *YAP3* | bZIP | fluconazole resistance |
| 19 | *FGR15* | *RPN4* | Zinc finger | filamentous growth |
| 20 | *FGR17* | *CHA4* | Zinc cluster | filamentous growth |
| 21 | *FKH2* | *FKH2* | Forkhead-like | filamentous growth and virulence |
| 22 | *FLO8* | *FLO8* | LUFS domain | filamentous growth and virulence |
| 23 | *GAL4* | *GAL4* | Zinc cluster | carbohydrate metabolic process |
| 24 | *GAT2* | *GAT2* | Zinc finger | filamentous growth |
| 25 | *GCF1* | N/A | HMG-like | binding to the promoter of *HWP1*, a biofilm-associated gene |
| 26 | *GCN4* | *GCN4* | bZIP | filamentous growth induced upon amino acid starvation |
| 27 | *GLN3* | *GLN3* | Zinc finger | filamentous growth |
| 28 | *HAC1* | *HAC1* | bZIP | filamentous growth |
| 29 | *HAL9* | *HAL9* | Zinc cluster | stress response and salt stress tolerance |
| 30 | *HAP31* | *HAP3* | Histone-like | carbohydrate metabolic process and iron-regulated |
| 31 | *HAP43* | *YAP3* | bZIP | stress response and iron limitation regulation |
| 32 | *HAP5* | *HAP5* | Histone-like | filamentous growth |
| 33 | *IRO1* | *YJL225C* | Unknown | filamentous growth and pathogenesis |
| 34 | *LYS14* | *LYS14* | Zinc cluster | amino acid metabolic process |
| 35 | *MAC1* | *MAC1* | Copper-first domain | filamentous growth |
| 36 | *MCM1* | *MCM1* | MADS domain | filamentous growth |
| 37 | *MDM34* | *MDM34* | Zinc finger | a putative virulence gene, macrophage-downregulated |
| 38 | *MIG1* | *MIG1* | Zinc finger | hyphal growth and upregulated during biofilm formation |
| 39 | *MNL1* | *MSN2* | Zinc finger | stress response |
| 40 | *MSN4* | *MSN4* | Zinc finger | stress response |
| 41 | *NDT80* | *NDT80* | PhoG-like | drug resistance |
| 42 | *NHP6A* | *NHP6A* | Unknown | anti-fungal drug regulated |
| 43 | *NOT3* | *NOT3* | Unknown | filamentous growth |
| 44 | *NOT5* | *NOT5* | Unknown | filamentous growth, adhesion and pathogenesis |
| 45 | *NRG1* | *YPR015C* | Zinc finger | filamentous growth and virulence |
| 46 | *RBF1* | *DEF1* | Unknown | filamentous growth |
| 47 | *RIM101* | *RIM101* | Zinc finger | filamentous growth |
| 48 | *RIM13* | *RIM13* | Unknown | filamentous growth |
| 49 | *RIM8* | *RIM8* | Unknown | filamentous growth |
| 50 | *RLM1* | *RLM1* | MEF2_like | resistance to cell wall stress |
| 51 | *SPT14* | *SPT14* | Unknown | cell wall biogenesis and upregulated in biofilm |
| 52 | *SPT20* | *SPT20* | Unknown | adherence to polystyrene |
| 53 | *STB5* | *STB5* | Zinc cluster | stress response |
| 54 | *STP3* | *STP2* | Zinc finger | RNA metabolic process |
| 55 | *STP4* | *STP4* | Zinc finger | RNA metabolic process |
| 56 | *TAF14* | *TAF14* | TATA binding | a putative virulence gene, macrophage induced |
| 57 | *TEA1* | *TEA1* | Zinc cluster | drug resistance |
| 58 | *TEC1* | *TEC1* | TEA domain | biofilm formation and filamentous growth |
| 59 | *TFG1* | *TFG1* | TFIIFa | filamentous growth |
| 60 | *THI20* | *THI20* | TENA | thiamine biosynthesis |
| 61 | *TYE7* | *TYE7* | bHLH | filamentous growth and drug resistance |
| 62 | *UGA3* | *UGA3* | Zinc cluster | gamma-aminobutyrate metabolism |
| 63 | *UGA32* | *UGA3* | Zinc finger | gamma-aminobutyrate metabolism |
| 64 | *UGA33* | *UGA3* | Zinc finger | gamma-aminobutyrate metabolism |
| 65 | *UPC2* | *UPC2* | Zinc cluster | cell wall biosynthesis |
| 66 | *ZCF5* | *HAP1* | Zinc cluster | carbohydrate metabolic process |
| 67 | *ZCF6* | *ASG1* | Zinc cluster | inferred function in stress response and drug resistance |
| 68 | *ZCF9* | *LYS14* | Zinc cluster | response to drug and up-regulated by pheromone |
| 69 | *ZCF11* | *YBR239C* | Zinc cluster | filamentous growth |
| 70 | *ZCF12* | *ECM22* | Zinc cluster | cell wall biosynthesis |
| 71 | *ZCF14* | *HAP1* | Zinc cluster | caspofungin induced |
| 72 | *ZCF16* | *CAT8* | Zinc finger | multidrug resistance and aerobic growth |
| 73 | *ZCF17* | *UPC2* | Zinc cluster | cell wall biosynthesis |
| 74 | *ZCF21* | *MUC1* | Zinc finger | invasive growth, flocculation and biofilms |
| 75 | *ZCF22* | *UPC2* | Zinc finger | cell wall biosynthesis |
| 76 | *ZCF23* | *GSM1* | Zinc finger | inferred function in energy metabolism |
| 77 | *ZCF24* | *ASG1* | Zinc finger | stress response |
| 78 | *ZCF28* | *ECM22* | Zinc cluster | cell wall biosynthesis |
| 79 | *ZCF32* | *LYS14* | Zinc finger | amino acid metabolic process |
| 80 | *ZCF38* | *TEA1* | Zinc cluster | stress response |
| 81 | *ZCF39* | *STB5* | Zinc cluster | induced during filamentation |
| 82 | *ZPR1* | *ZPR1* | Zinc finger | drug resistance |
| 83 | *19.1007* | *YDR017C* | bZIP | stress response and telomere maintenance |
| 84 | *19.1178* | Unknown | bZIP | unknown function |
| 85 | *19.1757* | *MET32* | Zinc finger | amino acid metabolic process |
| 86 | *19.226* | *BCD1* | Zinc finger | filamentous growth |
| 87 | *19.2315* | *RTG3* | bZIP | RNA metabolic process |
| 88 | *19.2393* | *YTH1* | Zinc finger | RNA metabolic process |
| 89 | *19.2399* | *YNL227C* | Zinc finger | ribosome biogenesis |
| 90 | *19.2458* | *SIP5* | Zinc finger | stress response |
| 91 | *19.2612* | *SWI5* | Zinc finger | RNA metabolic process |
| 92 | *19.2961* | *MIG2* | Zinc finger | inferred function in resistance to chemicals |
| 93 | *19.3088* | Unknown | bZIP | biofilm formation and hyphal formation |
| 94 | *19.3407* | *RAD18* | Zinc finger | stress response |
| 95 | *19.3683* | *GCS1* | Zinc finger | stress response |
| 96 | *19.3928* | *AZF1* | Zinc finger | response to chemical stimulus |
| 97 | *19.4125* | *PZF1* | Zinc finger | RNA metabolic process |
| 98 | *19.4778* | *LYS14* | Zinc cluster | lysine biosynthesis |
| 99 | *19.4972* | *CRZ1* | Zinc finger | response to chemical stimulus |
| 100 | *19.4998* | *TEA1* | Zinc cluster | amino acid metabolic process |
| 101 | *19.5326* | *MIG1* | Zinc finger | carbohydrate metabolic process |
| 102 | *19.5953* | *SFP1* | Zinc finger | RNA metabolic process |
| 103 | *19.5975* | *ADR1* | Zinc finger | fluconazole-downregulated |
| 104 | *19.6781* | *LYS14* | Zinc finger | amino acid metabolic process |
| 105 | *19.684* | *PCF11* | Zinc finger | response to drug |
| 106 | *19.6845* | Unknown | bZIP | unknown function |
| 107 | *19.6888* | *YLLO54C* | Gal4p-like | drug resistance |
