# Supplementary material for: Tec1 Mediates the Pheromone Response of the White Phenotype of Candida albicans: Insights into the Evolution of New Signal Transduction Pathways
Source: PLoS Biol. 2010 May 4;8(5):e1000363. doi: 10.1371/journal.pbio.1000363 (PMC2864266; doi:10.1371/journal.pbio.1000363)
Supplement: Table S2 — Oligonucleotides used for library construction in this study. (0.22 MB DOC) [file pbio.1000363.s005.doc]

| **Supporting information** | |  | |
| --- | --- | --- | --- |
|  |  |  |  |
| **Supplemental Table S2. Oligonucleotides used for library construction in this study** | | | |
|  |  |  |  |
| **Gene/ORF** | **Primer** | **Sequence** | |
| *ACE2* | ACE2f | 5'-TCCGTCGACAAAGATGCATTGGAAATTTCTG-3' | |
|  | ACE2r | 5'-TCCGTCGACAATTGCAACATTAAAAACTC-3' | |
| *ADA2* | ADA2f | 5’-TCCGTCGACAAAGATGGATTCAAGAACAAAA-3’ | |
|  | ADA2r | 5’-TCCGTCGACAACCCCTGAGAACACCATCCCA-3’ | |
| *ASH1* | ASH1f | 5'-TCCGTCGACAAAGATGAGTTTAGTCCAGTCA-3' | |
|  | ASH1r | 5'-TCCGTCGACAAAGATTTAGGAAGTACTTC-3' | |
| *BCR1* | BCR1f | 5'-TCCGTCGACAAAGATGTCAGGGACATCACAA-3' | |
|  | BCR1r | 5'-TCCGTCGACAATTGTGATATTAAATTATT-3' | |
| *BDF1* | BDF1f | 5'-TCCGTCGACAAAGATGAATGCTGGCGACAAA-3' | |
|  | BDF1r | 5'-TCCGTCGACAACTCTTCTTCTGAACTTTC-3' | |
| *BRE1* | BRE1f | 5'-TCCGTCGACAAAGATGGCTGTTGATAACGAA-3' | |
|  | BRE1r | 5'-TCCGTCGACAACAAGTGAATTGATAACAA-3' | |
| *CAP1* | CAP1f | 5'-TCCGTCGACAAAGATGACAGATATTAAAAGA-3' | |
|  | CAP1r | 5'-TCCGTCGACAAATGTTTTATACTTCGCTC-3' | |
| *CAS5* | CAS5f | 5'-TCCGTCGACAAAGATGGAGAATTATTTATTA-3' | |
|  | CAS5r | 5'-TCCGTCGACAAGGAAACTTCTTTGTTTTC-3' | |
| *CRZ1* | CRZ1f | 5'-TCCGTCGACAAAGATGTCTAACAATCCTCAT-3' | |
|  | CRZ1r | 5'-TCCGTCGACAAAGTAATTTCAACACCACT-3' | |
| *CRZ2* | CRZ2f | 5'-TCCCTCGAGAAAGATGTTATCAACCATGTCT-3' | |
|  | CRZ2r | 5'-TCCCTCGAGAATTTATTAGATTGTAATAA-3' | |
| *CSR1* | CSR1f | 5'-TCCGTCGACAAAGATGGAACCTATTTCTAAAT-3' | |
|  | CSR1r | 5'-TCCGTCGACAATTTCTCAACTGAATGTTC-3' | |
| *CTA4* | CTA4f | 5'-TCCGTCGACAAAGATGACATCTGAACATAAA-3' | |
|  | CTA4r | 5'-TCCGTCGACAATCCATAAAAATATCCATC-3' | |
| *CWT1* | CWT1f | 5'-TCCGTCGACAAAGATGTCTACCATGAGTACT-3' | |
|  | CWT1r | 5'-TCCGTCGACAAAGGATCAATGGGGATAAA-3' | |
| *EFG1* | EFG1f | 5'-TCCGTCGACAAAGATGTCAACGTATTCTATA-3' | |
|  | EFG1r | 5'-TCCGTCGACAAATGACTGAACTTGGGGTG-3' | |
| *EFH1* | EFH1f | 5'-TCCGTCGACAAAGATGAATGGTATTATGACG-3' | |
|  | EFH1r | 5'-TCCGTCGACAATAATGTTTTGTGAACAGT-3' | |
| *FCR1* | FCR1f | 5'-TCCGTCGACAAAGATGTCTGACGATCATTCA-3' | |
|  | FCR1r | 5'-TCCGTCGACAAAATATTGAAGAAAGGATC-3' | |
| *FCR3* | FCR3f | 5’-TCCGTCGACAAAGATGAATTTTAAGACAGAAAATTC-3’ | |
|  | FCR3r | 5’-TCCGTCGACAAATTCAAACTACTTTCAATTGCCT-3’ | |
| *FGR15* | FGR15f | 5'-TCCGTCGACAAAGATGGAATCCACATTAAGT-3' | |
|  | FGR15r | 5'-TCCGTCGACAACTTATTGAAAGTTACTTC-3' | |
| *FGR17* | FGR17f | 5'-TCCGTCGACAAAGATGCTGTCAAAATCTAGA-3' | |
|  | FGR17r | 5'-TCCGTCGACAATAACATATCAAGTATGCC-3' | |
| *FKH2* | FKH2f | 5'-TCCGTCGACAAAGATGTCAGCACAATTTATC-3' | |
|  | FKH2r | 5'-TCCGTCGACAACAGATCAATCATTTCAGT-3' | |
| *FLO8* | FLO8f | 5'-TCCGTCGACAAAGATGGTTCCCAACACAACT-3' | |
|  | FLO8r | 5'-TCCGTCGACAAATCGCCATTTTCAATTGG-3' | |
| *GAL4* | GAL4f | 5'-TCCGTCGACAAAGATGTCTGAAACTAATGAA-3' | |
|  | GAL4r | 5'-TCCGTCGACAAAACGTTAACAGTTTCATC-3' | |
| *GAT2* | GAT2f | 5'-TCCGTCGACAAAGATGTCCAGTTCATCATCT-3' | |
|  | GAT2r | 5'-TCCGTCGACAAACATATGGTTGTTTGTTG-3' | |
| *GCF1* | GCF1f | 5’-TCCGTCGACAAAGATGTTGAGATCATTTGTA-3’ | |
|  | GCF1r | 5’-TCCGTCGACAAAAAGTCATCCTCCACTTT-3’ | |
| *GCN4* | GCN4f | 5’-TCCGTCGACAAAGATGCCTGCTACTACTCCT-3’ | |
|  | GCN4r | 5’-TCCGTCGACAAAAATTGAATACCATTAACTCTTA-3’ | |
| *GLN3* | GLN3f | 5'-TCCCTCGAGAAAGATGACTACATCGAATAGT-3' | |
|  | GLN3r | 5'-TCCCTCGAGAAAATGTCAAACTTCAACCA-3' | |
| *HAC1* | HAC1f | 5'-TCCGTCGACAAAGATGGAGTTAACTGTTGAT-3' | |
|  | HAC1r | 5'-TCCGTCGACAAGACTTTATGAACTTCAAC-3' | |
| *HAL9* | HAL9f | 5’-TCCGTCGACAAAGATGGATCCTGCTTATGAT-3’ | |
|  | HAL9r | 5’-TCCGTCGACAAGTTATAAAATATATCAGG-3’ | |
| *HAP31* | HAP31f | 5’-TCCGTCGACAAAGATGAATCAACAAAACGCA-3’ | |
|  | HAP31r | 5’-TCCGTCGACAACTTCCTGGCTTCTCGGTA-3’ | |
| *HAP43* | HAP43f | 5'-TCCGTCGACAAAGATGCCCGCAAAAGGTCCT-3' | |
|  | HAP43r | 5'-TCCGTCGACAAATTATATGCTCTTCTATC-3' | |
| *HAP5* | HAP5f | 5'-TCCGTCGACAAAGATGAACGAAGATCCACAG-3' | |
|  | HAP5r | 5'-TCCGTCGACAAATAATTGTTTTGGTAACC-3' | |
| *IRO1* | IRO1f | 5'-TCCGTCGACAAAGATGTTGGATAGATTAAAT-3' | |
|  | IRO1r | 5'-TCCGTCGACAAGTTCAAACTGTTTAAATA-3' | |
| *LYS14* | LYS14f | 5’-TCCGTCGACAAAGATGTCACAATCACCATCT-3’ | |
|  | LYS14r | 5’-TCCGTCGACAAGTATATCAATGTATCATC-3’ | |
| *MAC1* | MAC1f | 5’-TCCGTCGACAAAGATGATACTAATAGATGAT-3’ | |
|  | MAC1r | 5’-TCCGTCGACAATTTGGTCTTTTTTGAGCAA-3’ | |
| *MCM1* | MCM1f | 5'-TCCGTCGACAAAGATGGCTATTAAGAAGAA-3' | |
|  | MCM1r | 5'-TCCGTCGACAATTGATATTGCTGTTGATT-3' | |
| *MDM34* | MDM34f | 5'-TCCCTCGAGAAAGATGTCGTTCAAAGTAAAT-3' | |
|  | MDM34r | 5'-TCCCTCGAGAAACAATATGGTGGTGGTGG-3' | |
| *MIG1* | MIG1f | 5'-TCCGTCGACAAAGATGTCCATGTCCACACCT-3' | |
|  | MIG1r | 5'-TCCGTCGACAAACTTAATAAATTGGTTAA-3' | |
| *MNL1* | MNL1f | 5'-TCCGTCGACAAAGATGGATTCACATAATAAC-3' | |
|  | MNL1r | 5'-TCCGTCGACAATCCTGAAGCATCATCCAT-3' | |
| *MSN4* | MSN4f | 5'-TCCGTCGACAAAGATGTCTCAAGAATTCCAA-3' | |
|  | MSN4r | 5'-TCCGTCGACAATACCGATTTTTTCTTTTC-3' | |
| *NDT80* | NDT80f | 5'-TCCCTCGAGAAAGATGCATCCATCAGCTGGT-3' | |
|  | NDT80r | 5'-TCCCTCGAGAACTGTGGAGGAGTAGGGGT-3' | |
| *NHP6A* | NHP6Af | 5’-TCCGTCGACAACACTTTGCATTTTCTGAT-3’ | |
|  | NHP6Ar | 5’-TCCGTCGACAAGGCGGAATTCTTTTTAGC-3’ | |
| *NOT3* | NOT3f | 5’-TCCGTCGACAAAGATGTCAAATCGAAAACTA-3’ | |
|  | NOT3r | 5’-TCCGTCGACAAAAATAATGTTTTTGATGG-3’ | |
| *NOT5* | NOT5f | 5'-TCCGTCGACAAAGATGAGTGCAAGAAAACTA-3' | |
|  | NOT5r | 5'-TCCGTCGACAATTGGAAAATCTGTCTGTT-3' | |
| *NRG1* | NRG1f | 5'-TCCGTCGACAAAGATGCTTTATCAACAATCA-3' | |
|  | NRG1r | 5'-TCCGTCGACAATACTAGGCTCTTGGTGTTG-3' | |
| *RBF1* | RBF1f | 5'-TCCGTCGACAAAGATGTCATCTAATAAGAAC-3' | |
|  | RBF1r | 5'-TCCGTCGACAACAAAAACCCACTTCTTTT-3' | |
| *RIM101* | RIM101f | 5'-TCCGTCGACAAAGATGAATTACAACATTCAT-3' | |
|  | RIM101r | 5'-TCCGTCGACAAGAAAGCAGTTATAGTTGG-3' | |
| *RIM13* | RIM13f | 5’-TCCGTCGACAAAGATGCCCACACCATGTCTA-3’ | |
|  | RIM13r | 5’-TCCGTCGACAATTTTAATATCACTTTATTATTGCAGC-3’ | |
| *RIM8* | RIM8f | 5’-TCCGTCGACAAAGATGAGACGAGCAGTATCAA-3’ | |
|  | RIM8r | 5’-TCCGTCGACAACGTTCTCTGAATTCGAGTTATT-3’ | |
| *RLM1* | RLM1f | 5'-TCCCTCGAGAAAGATGGGTAGAAGAAAGATT-3' | |
|  | RLM1r | 5'-TCCCTCGAGAATGTATTTTTATTAGGTCC-3' | |
| *SPT14* | SPT14f | 5'-TCCGTCGACAAAGATGGGATACAATATAGCA-3' | |
|  | SPT14r | 5'-TCCGTCGACAAATTTACTTTGTTCGGAAA-3' | |
| *SPT20* | SPT20f | 5'-TCCGTCGACAAAGATGATAAAATCTGAAGTT-3' | |
|  | SPT20r | 5'-TCCGTCGACAAATTAGCAGGCGCATTTTT-3' | |
| *STB5* | STB5f | 5'-TCCGTCGACAAAGATGAGACCAATAGACTCC-3' | |
|  | STB5r | 5'-TCCGTCGACAAAAAATTGTACATGAAATC-3' | |
| *STP3* | STP3f | 5'-TCCGTCGACAAAGATGTTGATACTTTCCATA-3' | |
|  | STP3r | 5'-TCCGTCGACAAATCTAGTAATAGATTGCT-3' | |
| *STP4* | STP4f | 5'-TCCGTCGACAAAGATGTTATCAATGGCCGTA-3' | |
|  | STP4r | 5'-TCCGTCGACAAATGTTCTTTTTTGATCAA-3' | |
| *TAF14* | TAF14f | 5'-TCCGTCGACAAAGATGTCAGAAGTAAAAAGG-3' | |
|  | TAF14r | 5'-TCCGTCGACAAAGCTTCACCAGTGTGTTT-3' | |
| *TEA1* | TEA1f | 5’-TCCGTCGACAAAGATGTCAATCAATTCATCA-3’ | |
|  | TEA1r | 5’-TCCGTCGACAAATTTTTCGAATTAAATAT-3’ | |
| *TEC1* | TEC1f | 5'-TCCGTCGACAAAGATGATGTCGCAAGCTACT-3' | |
|  | TEC1r | 5'-TCCGTCGACAAAAACTCACTAGTAAATCC-3' | |
| *TFG1* | TFG1f | 5'-TCCGTCGACAAAGATGAGTCAATCAGATGTT-3' | |
|  | TFG1r | 5'-TCCGTCGACAAGTCTTTAAGAACTAGCTT-3' | |
| *THI20* | THI20f | 5'-TCCCTCGAGAAAGATGACAATTGCTGGTAGC-3' | |
|  | THI20r | 5'-TCCCTCGAGAATATGTTCAACACTTCATC-3' | |
| *TYE7* | TYE7f | 5'-TCCGTCGACAAAGATGAGTTCATTCCAGCAA-3' | |
|  | TYE7r | 5'-TCCGTCGACAATATTTCACCACCCAATTT-3' | |
| *UGA3* | UGA3f | 5'-TCCGTCGACAAAGATGATAGTAACATTTAAT-3' | |
|  | UGA3r | 5'-TCCGTCGACAATGCAAAATTTATATCCCA-3' | |
| *UGA32* | UGA32f | 5'-TCCGTCGACAAAGATGTTCTACGTATTCGAT-3' | |
|  | UGA32r | 5'-TCCGTCGACAAGCAAAATGAGATGTTCC-3' | |
| *UGA33* | UGA33f | 5'-TCCGTCGACAAAGATGTCCTCACAATCCCCA-3' | |
|  | UGA33r | 5'-TCCGTCGACAAAATCATGGATATTTTCCA-3' | |
| *UPC2* | UPC2f | 5'-TCCCTCGAGAAAGATGATGATGACAGTGAAA-3' | |
|  | UPC2r | 5'-TCCCTCGAGAATTTCATATTCATAAACCC-3' | |
| *ZCF5* | ZCF5f | 5'-TCCGTCGACAAAGATGGAACTTGAAGCTAGT-3' | |
|  | ZCF5r | 5'-TCCGTCGACAATTTACTCTCTCTATATATTT-3' | |
| *ZCF6* | ZCF6f | 5’-TCCGTCGACAAAGATGAGTCAAGATCAAACCCCA-3’ | |
|  | ZCF6r | 5’-TCCGTCGACAATACACATAATTGTTTTGAATTGGCAA-3’ | |
| *ZCF9* | ZCF9f | 5'-TCCGTCGACAAAGATGCCTCTCGATAATACT-3' | |
|  | ZCF9r | 5'-TCCGTCGACAACCCGAGTAGCACCTCCCA-3' | |
| *ZCF11* | ZCF11f | 5'-TCCGTCGACAAAGATGAAGATTAAACAGGAA-3' | |
|  | ZCF11r | 5'-TCCGTCGACAATAGTATTGGTAAAAAGTT-3' | |
| *ZCF12* | ZCF12f | 5'-TCCCTCGAGAAAGATGGGAGACTCGCCTCCA-3' | |
|  | ZCF12r | 5'-TCCCTCGAGAATTGCCTGGGATCAAAATC-3' | |
| *ZCF14* | ZCF14f | 5'-TCCGTCGACAAAGATGCCAATAACAAAAAAC-3' | |
|  | ZCF14r | 5'-TCCGTCGACAATTTTTTCAATTGTGCCAA-3' | |
| *ZCF16* | ZCF16f | 5’-TCCGTCGACAAAGATGTCAAAGAAAAATAAAAAATCT-3’ | |
|  | ZCF16r | 5’-TCCGTCGACAAATATTGGGGACTTTGAGA-3’ | |
| *ZCF17* | ZCF17f | 5’-TCCGTCGACAAAGATGACGAAAACTACAGTC-3’ | |
|  | ZCF17r | 5’-TCCGTCGACAATCTATTCAGCGAAAACAA-3’ | |
| *ZCF21* | ZCF21f | 5'-TCCGTCGACAAAGATGATGGATATTTATCAG-3' | |
|  | ZCF21r | 5'-TCCGTCGACAAAGTGATCAATTTGGAAAT-3' | |
| *ZCF22* | ZCF22f | 5'-TCCGTCGACAAAGATGTATTGTGGATACTAT-3' | |
|  | ZCF22r | 5'-TCCGTCGACAAAAAGGCGACACTTTCGA-3' | |
| *ZCF23* | ZCF23f | 5’-TCCGTCGACAAAGATGACTAAAAAGTTAACT-3’ | |
|  | ZCF23r | 5’-TCCGTCGACAATACAATTGGCAAGAATTG-3’ | |
| *ZCF24* | ZCF24f | 5'-TCCCTCGAGAAAGATGCCAATGAGAAATAGA-3' | |
|  | ZCF24r | 5'-TCCCTCGAGAATATATCCAGCCATTTCTG-3' | |
| *ZCF28* | ZCF28f | 5'-TCCGTCGACAAAGATGAATCAAGATTCAACG-3' | |
|  | ZCF28r | 5'-TCCGTCGACAAATTTATTCCTTCACGACC-3' | |
| *ZCF32* | ZCF32f | 5'-TCCGTCGACAAAGATGGAGGAAAAGAAGAAA-3' | |
|  | ZCF32r | 5'-TCCGTCGACAACAACAATGTTAGATCAAC-3' | |
| *ZCF38* | ZCF38f | 5’-TCCCTCGAGAAAGATGTCAAATTCAACAACT-3’ | |
|  | ZCF38r | 5’-TCCCTCGAGTATGTTCATAGCATCATT-3’ | |
| *ZCF39* | ZCF39f | 5'-TCCGTCGACAAAGATGTCTACCGATACAACT-3' | |
|  | ZCF39r | 5'-TCCGTCGACAATGAAAATCTATTAAAATC-3' | |
| *ZPR1* | ZPR1f | 5’-TCCGTCGACAAAGATGTCTGAAGAAGGAGCTCATA-3’ | |
|  | ZPR1r | 5’-TCCGTCGACAAATCAGTTTTAATATCATTTAAACCTA-3’ | |
| *19.1007* | 19.1007f | 5’-TCCGTCGACAAAGATGGTGTTAATTGTAGTTGATGTA-3’ | |
|  | 19.1007r | 5’-TCCGTCGACAACTCTGAAAGTCCTTCGTCTTCT-3’ | |
| *19.1178* | 19.1178f | 5’-TCCGTCGACAAAGATGAACGAATTGTTTGATGCTA-3’ | |
|  | 19.1178r | 5’-TCCGTCGACAAACGACATAGATCAATCTCGA-3’ | |
| *19.1757* | 19.1757f | 5'-TCCGTCGACAAAGATGCAAAATACTAACCGT-3' | |
|  | 19.1757r | 5'-TCCGTCGACAAATTCTGTTGATATCCATA-3' | |
| *19.2260* | 19.2260f | 5’-TCCGTCGACAAAGATGGATTTTGAAGAAGAGACTA-3’ | |
|  | 19.2260r | 5’-TCCGTCGACAATGGTTCTTCTACAGTCTTGTTA-3’ | |
| *19.2315* | 19.2315f | 5’-TCCGTCGACAAAGATGGGAGATTACTTAAAC-3’ | |
|  | 19.2315r | 5’-TCCGTCGACAAGATATTCTCAGTACTAGTACCT-3’ | |
| *19.2393* | 19.2393f | 5’-TCCCTCGAGAAAGATGTCTTCACAAGTTCCA-3’ | |
|  | 19.2393r | 5’-TCCCTCGAGTAGAACTACATAATCTTC-3’ | |
| *19.2399* | 19.2399f | 5’-TCCGTCGACAAAGATGAAAACATGTTACTAT-3’ | |
|  | 19.2399r | 5’-TCCGTCGACAATTTGCGTTTATTTTTCTT-3’ | |
| *19.2458* | 19.2458f | 5’-TCCGTCGACAAAGATGGGTAATGTACCAGCT-3’ | |
|  | 19.2458r | 5’-TCCGTCGACAATTTTTTATTCTCTTTAT-3’ | |
| *19.2612* | 19.2612f | 5'-TCCGTCGACAAAGATGGTTAAACAGAAACAA-3' | |
|  | 19.2612r | 5'-TCCGTCGACAATGTATTTTCAGTGTTGTT-3' | |
| *19.2961* | 19.2961f | 5'-TCCGTCGACAAAGATGAGCAATCCAAACGAA-3' | |
|  | 19.2961r | 5'-TCCGTCGACAAACCACGTAACAACTCTTC-3' | |
| *19.3088* | 19.3088f | 5'-TCCCTCGAGAAAGATGGTATGTGCACATACT-3' | |
|  | 19.3088r | 5'-TCCCTCGAGAAAGCCAATGCTTTTCTTGA-3' | |
| *19.3407* | 19.3407f | 5’-TCCGTCGACAAAGATGAACCTCAAAGATATTACC-3’ | |
|  | 19.3407r | 5’-TCCGTCGACAAGTTACTCCGTGCTCTTGC-3’ | |
| *19.3683* | 19.3683f | 5’-TCCGTCGACAAAGATGTCCATTGATCCAGAAACT-3’ | |
|  | 19.3683r | 5’-TCCGTCGACAAAAAGTCATCCCATTTATCATCA-3’ | |
| *19.3928* | 19.3928f | 5’-TCCGTCGACAAAGATGACCTTATCATCAAGA-3’ | |
|  | 19.3928r | 5’-TCCGTCGACAAGAATGCCTCTCCTTTGGCTC-3’ | |
| *19.4125* | 19.4125f | 5'-TCCCTCGAGAAAGATGAGTGAAAGTGACGAA-3' | |
|  | 19.4125r | 5'-TCCCTCGAGAATCGAGAAATCACTGATGT-3' | |
| *19.4778* | 19.4778f | 5’-TCCGTCGACAAAGATGTCTACTTCCAAGAGA-3’ | |
|  | 19.4778r | 5’-TCCGTCGACAAGTAGGCAACATTATCGAT-3’ | |
| *19.4972* | 19.4972f | 5'-TCCGTCGACAAAGATGAATCTGAATTCTAAT-3' | |
|  | 19.4972r | 5'-TCCGTCGACAACAATGGTTTTTCATCACT-3' | |
| *19.4998* | 19.4998f | 5’-TCCGTCGACAAAGATGACACCAAGTTCAACT-3’ | |
|  | 19.4998r | 5’-TCCGTCGACAATAAACGAAATCCTTCTG-3’ | |
| *19.5326* | 19.5326f | 5'-TCCCTCGAGAAAGATGAGCATAGTAGACCAA-3' | |
|  | 19.5326r | 5'-TCCCTCGAGAATTTATTAAATTCAGGTAA-3' | |
| *19.5953* | 19.5953f | 5’-TCCCTCGAGATGTTTAATACCAAGATA-3’ | |
|  | 19.5953r | 5’-TCCCTCGAGATGAGTGGTATGCCCACG-3’ | |
| *19.5975* | 19.5975f | 5'-TCCCTCGAGAAAGATGTCTTTACCAATGTCA-3' | |
|  | 19.5975r | 5'-TCCCTCGAGAAACTGACCAACATATTA-3' | |
| *19.6781* | 19.6781f | 5’-TCCGTCGACAAAGATGTCTAAAAGAAGAACG-3’ | |
|  | 19.6781r | 5’-TCCGTCGACAAATTAACATCTAGTTCAGG-3’ | |
| *19.684* | 19.684f | 5’-TCCGTCGACAAAGATGACAGACATTTTGGAAGCAT-3’ | |
|  | 19.684r | 5’-TCCGTCGACAAATCATCATTGTCCGCTGGTCTCT-3’ | |
| *19.6845* | 19.6845f | 5’-TCCGTCGACAAAGATGGGATTTATTAATCCAGGAA-3’ | |
|  | 19.6845r | 5’-TCCGTCGACAAACCAAATCTATGTAGTATATCGT-3’ | |
| *19.6888* | 19.6888f | 5’-TCCCTCGAGATGGCAGCCAAGAAGGGA-3’ | |
|  | 19.6888r | 5’-TCCCTCGAGTATGCATTGTAGTAAAGT-3’ | |
