# Supplementary material for: Tec1 Mediates the Pheromone Response of the White Phenotype of Candida albicans: Insights into the Evolution of New Signal Transduction Pathways
Source: PLoS Biol. 2010 May 4;8(5):e1000363. doi: 10.1371/journal.pbio.1000363 (PMC2864266; doi:10.1371/journal.pbio.1000363)
Supplement: Table S3 — C. albicans strains used in this study. (0.18 MB DOC) [file pbio.1000363.s006.doc]

| **Supporting information** | | | | |  | |  |
| --- | --- | --- | --- | --- | --- | --- | --- |
|  | |  | | |  | |  |
| **Supplemental Table S3. *C. albicans* strains used in this study.** | | | | | | | |
|  | | | | | |  | |
| Strain | Parent | | *MTL* | Relevant Genotype | | Reference or source | |
| P37005 | − | | **a**/**a** | Wild type | | [1] | |
| WO-1 | − | | α/α | Wild type | | [2] | |
| *ste2/ste2* | P37005 | | **a**/**a** | *ste2*Δ::FRT/*ste2*Δ::FRT | | [3] | |
| *ste4/ste4* | P37005 | | **a**/**a** | *ste4*Δ::FRT/*ste4*Δ::FRT | | [3] | |
| *cek1/cek1 cek2/cek2* | P37005 | | **a**/**a** | *cek1*Δ::FRT/*cek1*Δ::FRT *cek2*Δ::FRT/*cek2*Δ::FRT | | [3] | |
| P37005-*TETp*-*STE11* | P37005 | | **a**/**a** | *ADH1*/*adh1*Δ::ptet-*STE11-GFP*::*SAT^R^* | | This study | |
| P37005-*TETp*-*CSH1* | P37005 | | **a**/**a** | *ADH1*/*adh1*Δ::ptet-*CSH1-GFP*::*SAT^R^* | | This study | |
| P37005-*TETp*-*PBR1* | P37005 | | **a**/**a** | *ADH1*/*adh1*Δ::ptet-*PBR1-GFP*::*SAT^R^* | | [4] | |
| P37005-*TETp*-*WH11* | P37005 | | **a**/**a** | *ADH1*/*adh1*Δ::ptet-*WH11-GFP*::*SAT^R^* | | This study | |
| *csh1/csh1* | P37005 | | **a**/**a** | *csh1*Δ::FRT/*csh1*Δ::FRT | | [4] | |
| *pbr1/pbr1* | P37005 | | **a**/**a** | *pbr1*Δ::FRT/*pbr1*Δ::FRT | | [4] | |
| *wh11/wh11* | P37005 | | **a**/**a** | *wh11*Δ::FRT/*wh11*Δ::FRT | | This study | |
| *tec1/TEC1* | P37005 | | **a**/**a** | *tec1*Δ::FRT/*TEC1* | | This study | |
| *tec1/tec1* | *tec1/TEC1* | | **a**/**a** | *tec1*Δ::FRT/*tec1*Δ::FRT | | This study | |
| *tec1/tec1-TEC1* | *tec1/tec1* | | **a**/**a** | *tec1*Δ::FRT/*tec1*Δ::FRT-*TEC1-GFP*::*SAT^R^* | | This study | |
| *tec1/TEC1-myc* | *tec1/TEC1* | | **a**/**a** | *tec1*Δ::FRT/*TEC1-myc*::*SAT^R^* | | This study | |
| L26-*TETp*-*TEC1* | L26 | | **a**/**a** | *ADH1*/*adh1*Δ::ptet-*TEC1-GFP*::*SAT^R^* | | This study | |
| P60002-*TETp*-*TEC1* | P60002 | | **a**/**a** | *ADH1*/*adh1*Δ::ptet-*TEC1-GFP*::*SAT^R^* | | This study | |
| WO-1-*TETp*-*TEC1* | WO-1 | | α/α | *ADH1*/*adh1*Δ::ptet-*TEC1-GFP*::*SAT^R^* | | This study | |
| 19F-*TETp*-*TEC1* | 19F | | α/α | *ADH1*/*adh1*Δ::ptet-*TEC1-GFP*::*SAT^R^* | | This study | |
| P57072-*TETp*-*TEC1* | P57072 | | α/α | *ADH1*/*adh1*Δ::ptet-*TEC1-GFP*::*SAT^R^* | | This study | |
| *ste2/ste2*-*TETp*-*TEC1* | *ste2/ste2* | | **a**/**a** | *ste2*Δ::FRT/*ste2*Δ::FRT *ADH1*/*adh1*Δ::ptet-*TEC1-GFP*::*SAT^R^* | | This study | |
| *ste4/ste4*-*TETp*-*TEC1* | *ste4/ste4* | | **a**/**a** | *ste4*Δ::FRT/*ste4*Δ::FRT *ADH1*/*adh1*Δ::ptet-*TEC1-GFP*::*SAT^R^* | | This study | |
| *cek1cek2*-*TETp*-*TEC1* | *cek1/cek1 cek2/cek2* | | **a**/**a** | *cek1*Δ::FRT/*cek1*Δ::FRT *cek2*Δ::FRT/*cek2*Δ::FRT *ADH1*/*adh1*Δ::ptet-*TEC1-GFP*::*SAT^R^* | | This study | |
| *tec1/tec1*-*TETp*-*TEC1* | *tec1/tec1* | | **a**/**a** | *tec1*Δ::FRT/*tec1*Δ::FRT *ADH1*/*adh1*Δ::ptet-*TEC1-GFP*::*SAT^R^* | | This study | |
| P37005-*TETp*-*ACE2* | P37005 | | **a**/**a** | *ADH1*/*adh1*Δ::ptet-*ACE2-GFP*::*SAT^R^* | | This study | |
| P37005-*TETp*-*ADA2* | P37005 | | **a**/**a** | *ADH1*/*adh1*Δ::ptet-*ADA2-GFP*::*SAT^R^* | | This study | |
| P37005-*TETp*-*ASH1* | P37005 | | **a**/**a** | *ADH1*/*adh1*Δ::ptet-*ASH1-GFP*::*SAT^R^* | | This study | |
| P37005-*TETp*-*BCR1* | P37005 | | **a**/**a** | *ADH1*/*adh1*Δ::ptet-*BCR1-GFP*::*SAT^R^* | | This study | |
| P37005-*TETp*-*BDF1* | P37005 | | **a**/**a** | *ADH1*/*adh1*Δ::ptet-*BDF1-GFP*::*SAT^R^* | | This study | |
| P37005-*TETp*-*BRE1* | P37005 | | **a**/**a** | *ADH1*/*adh1*Δ::ptet-*BRE1-GFP*::*SAT^R^* | | This study | |
| P37005-*TETp*-*CAP1* | P37005 | | **a**/**a** | *ADH1*/*adh1*Δ::ptet-*CAP1-GFP*::*SAT^R^* | | This study | |
| P37005-*TETp*-*CAS5* | P37005 | | **a**/**a** | *ADH1*/*adh1*Δ::ptet-*CAS5-GFP*::*SAT^R^* | | This study | |
| P37005-*TETp*-*CRZ1* | P37005 | | **a**/**a** | *ADH1*/*adh1*Δ::ptet-*CRZ1-GFP*::*SAT^R^* | | This study | |
| P37005-*TETp*-*CRZ2* | P37005 | | **a**/**a** | *ADH1*/*adh1*Δ::ptet-*CRZ2-GFP*::*SAT^R^* | | This study | |
| P37005-*TETp*-*CSR1* | P37005 | | **a**/**a** | *ADH1*/*adh1*Δ::ptet-*CSR1-GFP*::*SAT^R^* | | This study | |
| P37005-*TETp*-*CTA4* | P37005 | | **a**/**a** | *ADH1*/*adh1*Δ::ptet-*CTA4-GFP*::*SAT^R^* | | This study | |
| P37005-*TETp*-*CWT1* | P37005 | | **a**/**a** | *ADH1*/*adh1*Δ::ptet-*CWT1-GFP*::*SAT^R^* | | This study | |
| P37005-*TETp*-*EFG1* | P37005 | | **a**/**a** | *ADH1*/*adh1*Δ::ptet-*EFG1-GFP*::*SAT^R^* | | This study | |
| P37005-*TETp*-*EFH1* | P37005 | | **a**/**a** | *ADH1*/*adh1*Δ::ptet-*EFH1-GFP*::*SAT^R^* | | This study | |
| P37005-*TETp*-*FCR1* | P37005 | | **a**/**a** | *ADH1*/*adh1*Δ::ptet-*FCR1-GFP*::*SAT^R^* | | This study | |
| P37005-*TETp*-*FCR3* | P37005 | | **a**/**a** | *ADH1*/*adh1*Δ::ptet-*FCR3-GFP*::*SAT^R^* | | This study | |
| P37005-*TETp*-*FGR15* | P37005 | | **a**/**a** | *ADH1*/*adh1*Δ::ptet-*FGR15-GFP*::*SAT^R^* | | This study | |
| P37005-*TETp*-*FGR17* | P37005 | | **a**/**a** | *ADH1*/*adh1*Δ::ptet-*FGR17-GFP*::*SAT^R^* | | This study | |
| P37005-*TETp*-*FKH2* | P37005 | | **a**/**a** | *ADH1*/*adh1*Δ::ptet-*FKH2-GFP*::*SAT^R^* | | This study | |
| P37005-*TETp*-*FLO8* | P37005 | | **a**/**a** | *ADH1*/*adh1*Δ::ptet-*FLO8-GFP*::*SAT^R^* | | This study | |
| P37005-*TETp*-*GAL4* | P37005 | | **a**/**a** | *ADH1*/*adh1*Δ::ptet-*GAL4-GFP*::*SAT^R^* | | This study | |
| P37005-*TETp*-*GAT2* | P37005 | | **a**/**a** | *ADH1*/*adh1*Δ::ptet-*GAT2-GFP*::*SAT^R^* | | This study | |
| P37005-*TETp*-*GCF1* | P37005 | | **a**/**a** | *ADH1*/*adh1*Δ::ptet-*GCF1-GFP*::*SAT^R^* | | This study | |
| P37005-*TETp*-*GCN4* | P37005 | | **a**/**a** | *ADH1*/*adh1*Δ::ptet-*GCN4-GFP*::*SAT^R^* | | This study | |
| P37005-*TETp*-*GLN3* | P37005 | | **a**/**a** | *ADH1*/*adh1*Δ::ptet-*GLN3-GFP*::*SAT^R^* | | This study | |
| P37005-*TETp*-*HAC1* | P37005 | | **a**/**a** | *ADH1*/*adh1*Δ::ptet-*HAC1-GFP*::*SAT^R^* | | This study | |
| P37005-*TETp*-*HAL9* | P37005 | | **a**/**a** | *ADH1*/*adh1*Δ::ptet-*HAL9-GFP*::*SAT^R^* | | This study | |
| P37005-*TETp*-*HAP31* | P37005 | | **a**/**a** | *ADH1*/*adh1*Δ::ptet-*HAP31-GFP*::*SAT^R^* | | This study | |
| P37005-*TETp*-*HAP43* | P37005 | | **a**/**a** | *ADH1*/*adh1*Δ::ptet-*HAP43-GFP*::*SAT^R^* | | This study | |
| P37005-*TETp*-*HAP5* | P37005 | | **a**/**a** | *ADH1*/*adh1*Δ::ptet-*HAP5-GFP*::*SAT^R^* | | This study | |
| P37005-*TETp*-*IRO1* | P37005 | | **a**/**a** | *ADH1*/*adh1*Δ::ptet-*IRO1-GFP*::*SAT^R^* | | This study | |
| P37005-*TETp*-*LYS14* | P37005 | | **a**/**a** | *ADH1*/*adh1*Δ::ptet-*LYS14-GFP*::*SAT^R^* | | This study | |
| P37005-*TETp*-*MAC1* | P37005 | | **a**/**a** | *ADH1*/*adh1*Δ::ptet-*MAC1-GFP*::*SAT^R^* | | This study | |
| P37005-*TETp*-*MCM1* | P37005 | | **a**/**a** | *ADH1*/*adh1*Δ::ptet-*MCM1-GFP*::*SAT^R^* | | This study | |
| P37005-*TETp*-*MDM34* | P37005 | | **a**/**a** | *ADH1*/*adh1*Δ::ptet-*MDM34-GFP*::*SAT^R^* | | This study | |
| P37005-*TETp*-*MIG1* | P37005 | | **a**/**a** | *ADH1*/*adh1*Δ::ptet-*MIG1-GFP*::*SAT^R^* | | This study | |
| P37005-*TETp*-*MNL1* | P37005 | | **a**/**a** | *ADH1*/*adh1*Δ::ptet-*MNL1-GFP*::*SAT^R^* | | This study | |
| P37005-*TETp*-*MSN4* | P37005 | | **a**/**a** | *ADH1*/*adh1*Δ::ptet-*MSN4-GFP*::*SAT^R^* | | This study | |
| P37005-*TETp*-*NDT80* | P37005 | | **a**/**a** | *ADH1*/*adh1*Δ::ptet-*NDT80-GFP*::*SAT^R^* | | This study | |
| P37005-*TETp*-*NHP6A* | P37005 | | **a**/**a** | *ADH1*/*adh1*Δ::ptet-*NHP6A-GFP*::*SAT^R^* | | This study | |
| P37005-*TETp*-*NOT3* | P37005 | | **a**/**a** | *ADH1*/*adh1*Δ::ptet-*NOT3-GFP*::*SAT^R^* | | This study | |
| P37005-*TETp*-*NOT5* | P37005 | | **a**/**a** | *ADH1*/*adh1*Δ::ptet-*NOT5-GFP*::*SAT^R^* | | This study | |
| P37005-*TETp*-*NRG1* | P37005 | | **a**/**a** | *ADH1*/*adh1*Δ::ptet-*NRG1-GFP*::*SAT^R^* | | This study | |
| P37005-*TETp*-*RBF1* | P37005 | | **a**/**a** | *ADH1*/*adh1*Δ::ptet-*RBF1-GFP*::*SAT^R^* | | This study | |
| P37005-*TETp*-*RIM101* | P37005 | | **a**/**a** | *ADH1*/*adh1*Δ::ptet-*RIM101-GFP*::*SAT^R^* | | This study | |
| P37005-*TETp*-*RIM13* | P37005 | | **a**/**a** | *ADH1*/*adh1*Δ::ptet-*RIM13-GFP*::*SAT^R^* | | This study | |
| P37005-*TETp*-*RIM8* | P37005 | | **a**/**a** | *ADH1*/*adh1*Δ::ptet-*RIM8-GFP*::*SAT^R^* | | This study | |
| P37005-*TETp*-*RLM1* | P37005 | | **a**/**a** | *ADH1*/*adh1*Δ::ptet-*RLM1-GFP*::*SAT^R^* | | This study | |
| P37005-*TETp*-*SPT14* | P37005 | | **a**/**a** | *ADH1*/*adh1*Δ::ptet-*SPT14-GFP*::*SAT^R^* | | This study | |
| P37005-*TETp*-*SPT20* | P37005 | | **a**/**a** | *ADH1*/*adh1*Δ::ptet-*SPT20-GFP*::*SAT^R^* | | This study | |
| P37005-*TETp*-*STB5* | P37005 | | **a**/**a** | *ADH1*/*adh1*Δ::ptet-*STB5-GFP*::*SAT^R^* | | This study | |
| P37005-*TETp*-*STP3* | P37005 | | **a**/**a** | *ADH1*/*adh1*Δ::ptet-*STP3-GFP*::*SAT^R^* | | This study | |
| P37005-*TETp*-*STP4* | P37005 | | **a**/**a** | *ADH1*/*adh1*Δ::ptet-*STP4-GFP*::*SAT^R^* | | This study | |
| P37005-*TETp*-*TAF14* | P37005 | | **a**/**a** | *ADH1*/*adh1*Δ::ptet-*TAF14-GFP*::*SAT^R^* | | This study | |
| P37005-*TETp*-*TEA1* | P37005 | | **a**/**a** | *ADH1*/*adh1*Δ::ptet-*TEA1-GFP*::*SAT^R^* | | This study | |
| P37005-*TETp*-*TEC1* | P37005 | | **a**/**a** | *ADH1*/*adh1*Δ::ptet-*TEC1-GFP*::*SAT^R^* | | This study | |
| P37005-*TETp*-*TFG1* | P37005 | | **a**/**a** | *ADH1*/*adh1*Δ::ptet-*TFG1-GFP*::*SAT^R^* | | This study | |
| P37005-*TETp*-*THI20* | P37005 | | **a**/**a** | *ADH1*/*adh1*Δ::ptet-*THI20-GFP*::*SAT^R^* | | This study | |
| P37005-*TETp*-*TYE7* | P37005 | | **a**/**a** | *ADH1*/*adh1*Δ::ptet-*TYE7-GFP*::*SAT^R^* | | This study | |
| P37005-*TETp*-*UGA3* | P37005 | | **a**/**a** | *ADH1*/*adh1*Δ::ptet-*UGA3-GFP*::*SAT^R^* | | This study | |
| P37005-*TETp*-*UGA32* | P37005 | | **a**/**a** | *ADH1*/*adh1*Δ::ptet-*UGA32-GFP*::*SAT^R^* | | This study | |
| P37005-*TETp*-*UGA33* | P37005 | | **a**/**a** | *ADH1*/*adh1*Δ::ptet-*UGA33-GFP*::*SAT^R^* | | This study | |
| P37005-*TETp*-*UPC2* | P37005 | | **a**/**a** | *ADH1*/*adh1*Δ::ptet-*UPC2-GFP*::*SAT^R^* | | This study | |
| P37005-*TETp*-*ZCF5* | P37005 | | **a**/**a** | *ADH1*/*adh1*Δ::ptet-*ZCF5-GFP*::*SAT^R^* | | This study | |
| P37005-*TETp*-*ZCF6* | P37005 | | **a**/**a** | *ADH1*/*adh1*Δ::ptet-*ZCF6-GFP*::*SAT^R^* | | This study | |
| P37005-*TETp*-*ZCF9* | P37005 | | **a**/**a** | *ADH1*/*adh1*Δ::ptet-*ZCF9-GFP*::*SAT^R^* | | This study | |
| P37005-*TETp*-*ZCF11* | P37005 | | **a**/**a** | *ADH1*/*adh1*Δ::ptet-*ZCF11-GFP*::*SAT^R^* | | This study | |
| P37005-*TETp*-*ZCF12* | P37005 | | **a**/**a** | *ADH1*/*adh1*Δ::ptet-*ZCF12-GFP*::*SAT^R^* | | This study | |
| P37005-*TETp*-*ZCF14* | P37005 | | **a**/**a** | *ADH1*/*adh1*Δ::ptet-*ZCF14-GFP*::*SAT^R^* | | This study | |
| P37005-*TETp*-*ZCF16* | P37005 | | **a**/**a** | *ADH1*/*adh1*Δ::ptet-*ZCF16-GFP*::*SAT^R^* | | This study | |
| P37005-*TETp*-*ZCF17* | P37005 | | **a**/**a** | *ADH1*/*adh1*Δ::ptet-*ZCF17-GFP*::*SAT^R^* | | This study | |
| P37005-*TETp* -*ZCF21* | P37005 | | **a**/**a** | *ADH1*/*adh1*Δ::ptet-*ZCF21-GFP*::*SAT^R^* | | This study | |
| P37005-*TETp-ZCF22* | P37005 | | **a**/**a** | *ADH1*/*adh1*Δ::ptet-*ZCF22-GFP*::*SAT^R^* | | This study | |
| P37005-*TETp-ZCF23* | P37005 | | **a**/**a** | *ADH1*/*adh1*Δ::ptet-*ZCF23-GFP*::*SAT^R^* | | This study | |
| P37005-*TETp-ZCF24* | P37005 | | **a**/**a** | *ADH1*/*adh1*Δ::ptet-*ZCF24-GFP*::*SAT^R^* | | This study | |
| P37005-*TETp-ZCF28* | P37005 | | **a**/**a** | *ADH1*/*adh1*Δ::ptet-*ZCF28-GFP*::*SAT^R^* | | This study | |
| P37005-*TETp-ZCF32* | P37005 | | **a**/**a** | *ADH1*/*adh1*Δ::ptet-*ZCF32-GFP*::*SAT^R^* | | This study | |
| P37005-*TETp-ZCF38* | P37005 | | **a**/**a** | *ADH1*/*adh1*Δ::ptet-*ZCF38-GFP*::*SAT^R^* | | This study | |
| P37005-*TETp-ZCF39* | P37005 | | **a**/**a** | *ADH1*/*adh1*Δ::ptet-*ZCF39- GFP*::*SAT^R^* | | This study | |
| P37005-*TETp-ZPR1* | P37005 | | **a**/**a** | *ADH1*/*adh1*Δ::ptet-*ZPR1- GFP*::*SAT^R^* | | This study | |
| P37005-*TETp-1007* | P37005 | | **a**/**a** | *ADH1*/*adh1*Δ::ptet-*1007- GFP*::*SAT^R^* | | This study | |
| P37005-*TETp-1178* | P37005 | | **a**/**a** | *ADH1*/*adh1*Δ::ptet-*1178- GFP*::*SAT^R^* | | This study | |
| P37005-*TETp-1757* | P37005 | | **a**/**a** | *ADH1*/*adh1*Δ::ptet-*1757- GFP*::*SAT^R^* | | This study | |
| P37005-*TETp-2260* | P37005 | | **a**/**a** | *ADH1*/*adh1*Δ::ptet-*2260- GFP*::*SAT^R^* | | This study | |
| P37005-*TETp-2315* | P37005 | | **a**/**a** | *ADH1*/*adh1*Δ::ptet-*2315- GFP*::*SAT^R^* | | This study | |
| P37005-*TETp-2393* | P37005 | | **a**/**a** | *ADH1*/*adh1*Δ::ptet-*2393- GFP*::*SAT^R^* | | This study | |
| P37005-*TETp-2399* | P37005 | | **a**/**a** | *ADH1*/*adh1*Δ::ptet-*2399- GFP*::*SAT^R^* | | This study | |
| P37005-*TETp-2458* | P37005 | | **a**/**a** | *ADH1*/*adh1*Δ::ptet-*2458- GFP*::*SAT^R^* | | This study | |
| P37005-*TETp-2612* | P37005 | | **a**/**a** | *ADH1*/*adh1*Δ::ptet-*2612- GFP*::*SAT^R^* | | This study | |
| P37005-*TETp-2961* | P37005 | | **a**/**a** | *ADH1*/*adh1*Δ::ptet-*2961- GFP*::*SAT^R^* | | This study | |
| P37005-*TETp-3088* | P37005 | | **a**/**a** | *ADH1*/*adh1*Δ::ptet-*3088- GFP*::*SAT^R^* | | This study | |
| P37005-*TETp-3407* | P37005 | | **a**/**a** | *ADH1*/*adh1*Δ::ptet-*3407- GFP*::*SAT^R^* | | This study | |
| P37005-*TETp-3683* | P37005 | | **a**/**a** | *ADH1*/*adh1*Δ::ptet-*3683- GFP*::*SAT^R^* | | This study | |
| P37005-*TETp-3928* | P37005 | | **a**/**a** | *ADH1*/*adh1*Δ::ptet-*3928- GFP*::*SAT^R^* | | This study | |
| P37005-*TETp-4125* | P37005 | | **a**/**a** | *ADH1*/*adh1*Δ::ptet-*4125- GFP*::*SAT^R^* | | This study | |
| P37005-*TETp-4778* | P37005 | | **a**/**a** | *ADH1*/*adh1*Δ::ptet-*4778- GFP*::*SAT^R^* | | This study | |
| P37005-*TETp-4972* | P37005 | | **a**/**a** | *ADH1*/*adh1*Δ::ptet-*4972- GFP*::*SAT^R^* | | This study | |
| P37005-*TETp-4998* | P37005 | | **a**/**a** | *ADH1*/*adh1*Δ::ptet-*4998- GFP*::*SAT^R^* | | This study | |
| P37005-*TETp-5326* | P37005 | | **a**/**a** | *ADH1*/*adh1*Δ::ptet-*5326- GFP*::*SAT^R^* | | This study | |
| P37005-*TETp-5953* | P37005 | | **a**/**a** | *ADH1*/*adh1*Δ::ptet-*5953- GFP*::*SAT^R^* | | This study | |
| P37005-*TETp-5975* | P37005 | | **a**/**a** | *ADH1*/*adh1*Δ::ptet-*5975- GFP*::*SAT^R^* | | This study | |
| P37005-*TETp-6781* | P37005 | | **a**/**a** | *ADH1*/*adh1*Δ::ptet-*6781- GFP*::*SAT^R^* | | This study | |
| P37005-*TETp-684* | P37005 | | **a**/**a** | *ADH1*/*adh1*Δ::ptet-*684- GFP*::*SAT^R^* | | This study | |
| P37005-*TETp-6845* | P37005 | | **a**/**a** | *ADH1*/*adh1*Δ::ptet-*6845- GFP*::*SAT^R^* | | This study | |
| P37005-*TETp-6888* | P37005 | | **a**/**a** | *ADH1*/*adh1*Δ::ptet-*6888- GFP*::*SAT^R^* | | This study | |
|  |  | |  |  | |  | |
|  |  | |  |  | |  | |

References

1. Lockhart SR, Pujol C, Daniels KJ, Miller MG, Johnson AD, et al. (2002) In *Candida albicans*, white-opaque switchers are homozygous for mating type. Genetics 162: 737-745.
2. Slutsky B, Staebell M, Anderson J, Risen L, Pfaller M, et al. (1987) "White-opaque transition": a second high-frequency switching system in *Candida albicans*. J Bacteriol 169: 189-197.
3. Yi S, Sahni N, Daniels KJ, Pujol C, Srikantha T, et al. (2008) The same receptor, G protein, and mitogen-activated protein kinase pathway activate different downstream regulators in the alternative white and opaque pheromone responses of *Candida albicans*. Mol Biol Cell 19: 957-970.
4. Sahni N, Yi S, Pujol C, Soll DR (2009) The white cell response to pheromone is a general characteristic of *Candida albicans* strains. Eukaryot Cell 8: 251–256.
