# Supplementary material for: Tec1 Mediates the Pheromone Response of the White Phenotype of Candida albicans: Insights into the Evolution of New Signal Transduction Pathways
Source: PLoS Biol. 2010 May 4;8(5):e1000363. doi: 10.1371/journal.pbio.1000363 (PMC2864266; doi:10.1371/journal.pbio.1000363)
Supplement: Table S4 — List of genes used for MEME analysis. (0.05 MB DOC) [file pbio.1000363.s007.doc]

| **Supporting information** | |  |
| --- | --- | --- |
|  |  |  |
| **Supplemental Table S4. List of genes used for MEME analysis** | | |
|  |  |  |

| *C. albicans* WPRE [1] | *S. cerevisiae* TCS [2-4] | *C. albicans* OPRE [1] | *S. cerevisiae* PRE [3,5-7] |
| --- | --- | --- | --- |
| *CSH1* | *GSC2* | *MFA1* | *PRM1* |
| *PBR1* | *TIP1* | *FUS1* | *PRM3* |
| *RBT5* | *FLO11* | *CPH1* | *PRM6* |
| *WH11* | *CLN1* | *ECE1* | *FUS2* |
| *TEC1* | *TEC1* | *KAR4* | *CIK1* |
| *EAP1* | *PGU1* | *RAM1* | *PRM4* |
| *PGA10* | *SRL1* | *FGR23* | *AFR1* |
| *LSP1* | *CHS7* | *CAG1* | *AGA1* |
| *PHR1* | *PHD1* | *CEK1* | *SCW10* |
| *PHR2* | *CWP1* | *FIG1* | *ASG7* |
| *SUN41* | *FUS1* | *RBT4* | *FUS1* |
| *Orf19.2077* | *FUS3* | *HWP1* | *FUS3* |
| *CIT1* | *PRM2* | *ECE1* | *PRM2* |
| *STE2* | *PCL2* | *STE2* | *PCL2* |
| *CEK2* | *GIC2* | *CEK2* | *GIC2* |
| *SST2* | *SVS1* | *SST2* | *SVS1* |
| *RBT1* | *GFA1* | *RBT1* | *GFA1* |

References

1. Sahni N, Yi S, Daniels KJ, Srikantha T, Pujol C, et al. (2009) Genes selectively up-regulated by pheromone in white cells are involved in biofilm formation in *Candida albicans*. PLoS Pathog 5(10): e1000601. doi:10.1371/journal.ppat.1000601
2. Baur M, Esch RK, Errede B (1997) Cooperative binding interactions required for function of the Ty1 sterile responsive element. Mol Cell Biol 17: 4330-4337.
3. Madhani HD, Fink GR (1997) Combinatorial control required for the specificity of yeast MAPK signaling. Science 275: 1314–1317.
4. Chou S, Lane S, Liu H (2006) Regulation of mating and filamentation genes by two distinct Ste12 complexes in *Saccharomyces cerevisiae*. Mol Cell Biol 13: 4794-4805.
5. Dolan JW, Kirkman C, Fields S (1989) The yeast STE12 protein binds to the DNA sequence mediating pheromone induction. Proc Natl Acad Sci U S A 86: 5703–5707.
6. Errede B, Ammerer G (1989) STE12, a protein involved in cell-type-specific transcription and signal transduction in yeast, is part of protein-DNA complexes. Genes Dev 3: 1349–1361.
7. Hagen DC, McCaffrey G, Sprague GF Jr (1991) Pheromone response elements are necessary and sufficient for basal and pheromone-induced transcription of the FUS1 gene of *Saccharomyces cerevisiae*. Mol Cell Biol 11: 2952–2961.
