# Supplementary material for: Tec1 Mediates the Pheromone Response of the White Phenotype of Candida albicans: Insights into the Evolution of New Signal Transduction Pathways
Source: PLoS Biol. 2010 May 4;8(5):e1000363. doi: 10.1371/journal.pbio.1000363 (PMC2864266; doi:10.1371/journal.pbio.1000363)
Supplement: Table S5 — cis-Acting DNA motifs bound by Tec1 homologs in different species. (0.05 MB DOC) [file pbio.1000363.s008.doc]

| **Supporting information** | |  |
| --- | --- | --- |
|  |  |  |
| **Supplemental Table S5. *cis*-acting DNA motifs bound by Tec1 homologs in different species** | | |
|  |  |  |

| **Tec1 homologs** | **Organism** | **Target gene** | **Motif sequence** | **References** |
| --- | --- | --- | --- | --- |
| Tec1 | *Candida albicans* | *PHR2* | AAAAAAAAAAGAAAG | [1] |
|  |  | *RBT1* | AGAAAAAACAGAAAG | [1] |
|  |  | Consensus: | AAAAAAAAAAGAAAG | [1] |
| Tec1 | *Saccharomyces cerevisiae* | *SRL3* | AGAATG | [2] |
|  |  | *SVS1* | AGAATG | [2] |
|  |  | Consensus: | AGAATG | [2-4] |
| AbaA | *Aspergillus nidulans* | *wetA* | GGAATG | [5] |
|  |  | *rodA* | (G/A)GAATG | [5] |
|  |  | Consensus: | (G/A)GAATG | [5] |
| TEF-1 | *Homo sapiens* | GT-IIC | GGAATG | [6] |
|  |  | Sph-II | AGTATG | [6] |
|  |  | Consensus: | (G/A)G(A/T/C)ATG | [7-8] |

References

1. Sahni N, Yi S, Daniels KJ, Srikantha T, Pujol C, et al. (2009) Genes selectively up-regulated by pheromone in white cells are involved in biofilm formation in *Candida albicans*. PLoS Pathog 5(10): e1000601. doi:10.1371/journal.ppat.1000601
2. Chou S, Lane S, Liu H (2006) Regulation of mating and filamentation genes by two distinct Ste12 complexes in *Saccharomyces cerevisiae*. Mol Cell Biol 13: 4794-4805.
3. Baur M, Esch RK, Errede B (1997) Cooperative binding interactions required for function of the Ty1 sterile responsive element. Mol Cell Biol 17: 4330-4337.
4. Madhani HD, Fink GR (1997) Combinatorial control required for the specificity of yeast MAPK signaling. Science 275: 1314-1317.
5. Andrianopoulos A, Timberlake WE (1994) The *Aspergillus nidulans abaA* gene encodes a transcriptional activator that acts as a genetic switch to control development. Mol Cell Biol 14: 2503-2515.
6. Davidson I, Xiao JH, Rosales R, Staub A, Chambon P (1988) The HeLa cell protein TEF-1 binds specifically and cooperatively to two SV40 enhancer motifs of unrelated sequence. Cell 54: 931-942.
7. Hwang JJ, Chambon P, Davidson I (1993) Characterization of the transcription activation function and the DNA binding domain of transcriptional enhancer factor-1. EMBO J 12: 2337-2348.
8. Jacquemin P, Hwang JJ, Martial JA, Dolle P, Davidson I (1996) A novel family of developmentally regulated mammalian transcription factors containing the TEA/ATTS DNA binding domain. J Biol Chem 271: 21775-21785.
