# Supplementary material for: Tec1 Mediates the Pheromone Response of the White Phenotype of Candida albicans: Insights into the Evolution of New Signal Transduction Pathways
Source: PLoS Biol. 2010 May 4;8(5):e1000363. doi: 10.1371/journal.pbio.1000363 (PMC2864266; doi:10.1371/journal.pbio.1000363)
Supplement: Table S6 — Oligonucleotides used for mutant construction, Northern analysis, and ChIP-PCR. (0.08 MB DOC) [file pbio.1000363.s009.doc]

| **Supporting information** | |  |
| --- | --- | --- |
|  |  |  |
| **Supplemental Table S6. Oligonucleotides used for mutant construction, northern and ChIP-PCR** | | |
|  |  |  |
| **Primer** | **Gene/Purpose** | **Sequence** |
| TEC1f1 | *TEC1* heterozygote | 5'-TGTGTCTTGTGGTTAAGT-3' |
| TEC1r1 | *TEC1* heterozygote | 5'-TCCCCCGGGACAAATGTGAGATTGCAA-3' |
| TEC1f2 | *TEC1* heterozygote | 5'-TCCCCCGGGACTTACTCACTGTTGGAT-3' |
| TEC1r2 | *TEC1* heterozygote | 5'-TGATGCATTGAACAAGCT -3' |
| TEC1f3 | *TEC1* homozygote | 5'-ATGATGTCGCAAGCTACT -3' |
| TEC1r3 | *TEC1* homozygote | 5'-TCCCCCGGGTTCTGAATTTCCCGGTTT-3' |
| TEC1f4 | *TEC1* homozygote | 5'-TCCCCCGGGGAAAGTGAAGGTGGTCTTA-3' |
| TEC1r4 | *TEC1* homozygote | 5'-AAACTCACTAGTAAATCCT -3' |
| TEC1Q1f | *TEC1* complementation | 5’-TCCCCCGGGGAAAGTGAAGGTGGTCTTA-3’ |
| TEC1Q1r | *TEC1* complementation | 5’-TCCGGATCCAAACTCACTAGTAAATCCT-3’ |
| TEC1Q2f | *TEC1* complementation | 5’-TCCGGATCCACTTACTCACTGTTGGAT-3’ |
| TEC1Q2r | *TEC1* complementation | 5’- TGATGCATTGAACAAGCT-3’ |
| SATBgF1 | *GFP-SAT1* PCR | 5'-TCAAGATCTTCCATCATAAAATGTCGA-3' |
| GFBhF1 | *GFP-SAT1* PCR | 5'-TCAGGATCCATGTCTAAAGGTGAAGAA-3' |
| TEC1nf | Northern probe | 5'-ATGATGTCGCAAGCTACT-3' |
| TEC1nr | Northern probe | 5'-AAAACTCACTAGTAAATCC-3' |
| CEK1nf | Northern probe | 5'-CGTAGCTACAAGATGGTATAG-3' |
| CEK1nr | Northern probe | 5'-TCGTACCGCCAGTATTACTAG-3' |
| CEK2nf | Northern probe | 5'-GGGCTGTATATTGGCTGAACT-3' |
| CEK2nr | Northern probe | 5'-TCTAATGCGTCTTGAACGGTG-3' |
| CSH1nf | Northern probe | 5'-TCGACTCTGAAAAAACTA-3' |
| CSH1nr | Northern probe | 5'-CATGCCAATGAAACTTGC-3' |
| PBR1nf | Northern probe | 5’-AATGTGACTTTATACATT-3’ |
| PBR1nr | Northern probe | 5’-CAGCATATAAGTAATCAT-3’ |
| RBT5nf | Northern probe | 5'-TGATGCCGCTGCTGAAAC-3' |
| RBT5nr | Northern probe | 5'-ACAGCGGCAATGACACCA-3' |
| WH11nf | Northern probe | 5'-ATGTCCGACTTAGGTAGA-3' |
| WH11nr | Northern probe | 5'-TTATTTGGAGTCACCAAA-3' |
| KAR4nf | Northern probe | 5'-ATGTATACTTACAATAAGTTTGGG-3' |
| KAR4nr | Northern probe | 5'-TACCTCTGTAGCACCAGA-3' |
| MFA1nf | Northern probe | 5'-ATGGCTGCTCAACAACAA-3' |
| MFA1nr | Northern probe | 5'-TTACATAACAGAACAAGT-3' |
| STE2nf | Northern probe | 5'-GTGTTCAACATAAGAAGA-3' |
| STE2nr | Northern probe | 5'-ATTATTAGCAGTTTGAGC-3' |
| RBT1nf | Northern probe | 5'-AGCCACTGAATCAGTTCC-3' |
| RBT1nr | Northern probe | 5'-ATCAAGAATGCAGCAATACC-3' |
| ACT1nf | Northern probe | 5'-TTGGTGTTTGACGAGTTT-3' |
| ACT1nr | Northern probe | 5'-TACCGTGTTCAATTGGGTAT-3' |
| STE11f | MAPK hyperactivation | 5'-TCCGTCGACAAAGATGACAGAGATTAATGATT-3' |
| STE11r | MAPK hyperactivation | 5'-TCCGTCGACAATTGTTTCGACATAATTAATG-3' |
| TEC1mycf1 | TEC1-myc | 5’- TCCCCCGGGGAAAGTGAAGGTGGTCTTA-3' |
| TEC1mycr1 | TEC1-myc | 5’-TCCTCTAGAAAAGTGAAGGTGGTCTTA-3’ |
| TEC1mycf2 | TEC1-myc | 5’-TCCTCTAGAACTTACTCACTGTTGGAT-3’ |
| TEC1mycr2 | TEC1-myc | 5’- TGATGCATTGAACAAGCT-3’ |
| PBR1chpf | ChIP-PCR | 5’-TATCGCTCATACAATGATT-3’ |
| PBR1chpr | ChIP-PCR | 5’-TTTCAAGGAAGGAATGGA-3’ |
| CSH1chpf | ChIP-PCR | 5’-AGAGAACATTCAAGCTTG-3’ |
| CSH1chpr | ChIP-PCR | 5’-AGCAAAAACAGGCAGTAT-3’ |
| RBT5chpf | ChIP-PCR | 5’-AAGCCAAGCTGCATAAGTAT-3’ |
| RBT5chpr | ChIP-PCR | 5’-TGCCTATGTATTTATACCCT-3’ |
| WH11chpf | ChIP-PCR | 5’-TGTGGCACTTGATTTCTAGT-3’ |
| WH11chpr | ChIP-PCR | 5’-TTTAATTGTTCTGTTTGTTGTT-3’ |
| STE2chpf | ChIP-PCR | 5’-TACCCGTTTGATATTCAATT-3’ |
| STE2chpr | ChIP-PCR | 5’-AGTAAATCGTTTGGTGACGA-3’ |
| RBT1chpf | ChIP-PCR | 5’-TTGGGACCACGGTCATTCAA-3’ |
| RBT1chpr | ChIP-PCR | 5’-AACACGCCTTATAATGACAA-3’ |
| ACT1chpf | ChIP-PCR | 5’-TATTAAGTAGTGTGTGCACT-3’ |
| ACT1chpr | ChIP-PCR | 5’-TTGGCAATAAATCTTGGTGA-3’ |
| TEC1chpf | ChIP-PCR | 5’-TTCTCATTGCCTTAGTCA-3’ |
| TEC1chpf | ChIP-PCR | 5’-AATTAAGGGAAGTCAAGGT-3’ |
| KAR4chpf | ChIP-PCR | 5’-TTAGGGCGTTTCAGTGTTGT-3’ |
| KAR4chpr | ChIP-PCR | 5’-TGTTTCAAATATTTGGTGTTC-3’ |
| MFA1chpf | ChIP-PCR | 5’-TGTAGAGAACGTAAAGAGCT-3’ |
| MFA1chpr | ChIP-PCR | 5’-TATGCTCTATTTTTCGCAATT-3’ |
