# Supplementary material for: Tec1 Mediates the Pheromone Response of the White Phenotype of Candida albicans: Insights into the Evolution of New Signal Transduction Pathways
Source: PLoS Biol. 2010 May 4;8(5):e1000363. doi: 10.1371/journal.pbio.1000363 (PMC2864266; doi:10.1371/journal.pbio.1000363)
Supplement: Table S7 — Promoter comparative analysis using gene orthologs in C. albicans, C. dubliniensis, and C. tropicalis in MEME. (0.05 MB DOC) [file pbio.1000363.s010.doc]

| **Supporting information** | |  |
| --- | --- | --- |
|  |  |  |
| **Supplemental Table S7. Promoter comparative analysis using gene orthologs in *C. albicans*, *C. dubliniensis*, and *C. tropicalis* in MEME** | | |
|  |  |  |

| *C. albicans* ortholog | *C. dubliniensis* ortholog | *C. tropicalis* ortholog |
| --- | --- | --- |
| *CSH1* | *Cd36_03770* | *CTRG_04744* |
| *PBR1* | *Cd36_05950* | *CTRG_04251* |
| *RBT5* | *Cd36_40190* | *CTRG_00099* |
| *WH11* | *Cd36_19680* | *CTRG_01660* |
| *TEC1* | *Cd36_84480* | *CTRG_02294* |
| *EAP1* | *Cd36_23630* | *CTRG_02074* |
| *PGA10* | *Cd36_40510* | *CTRG_00109* |
| *LSP1* | *Cd36_21030* | *CTRG_01890* |
| *PHR1* | *Cd36_44230* | *CTRG_03942* |
| *PHR2* | *Cd36_00220* | *CTRG_04296* |
| *SUN41* | *Cd36_60800* | *CTRG_02944* |
| *Orf19.2077* | *Cd36_15470* | *CTRG_01124* |
| *CIT1* | *Cd36_28700* | *CTRG_00747* |
| *STE2* | *Cd36_32150* | *CTRG_00685* |
| *CEK2* | *Cd36_31320* | *CTRG_00659* |
| *SST2* | *Cd36_51980* | *CTRG_03200* |
| *RBT1* | *Cd36_43400* | *CTRG_00477* |
