# Supplementary material for: Tec1 Mediates the Pheromone Response of the White Phenotype of Candida albicans: Insights into the Evolution of New Signal Transduction Pathways
Source: PLoS Biol. 2010 May 4;8(5):e1000363. doi: 10.1371/journal.pbio.1000363 (PMC2864266; doi:10.1371/journal.pbio.1000363)
Supplement: Table S8 — Elicitation of cis-acting consensus regulatory motifs from the promoters of orthologs of Tec1-target genes in the three fungal species C. albicans, C. dubliniensis, and C. tropicalis. (0.10 MB DOC) [file pbio.1000363.s011.doc]

| **Supporting information** | |  |
| --- | --- | --- |
|  |  |  |
| **Table S8. Elicitation of *cis*-acting consensus regulatory motifs from the promoters of orthologs of Tec1-target genes in the three fungal species *C. albicans*, *C. dubliniensis*, *C. tropicalis*.** | | |

1. *C. albicans*:

| Gene | WPRE  position | WPRE | P value | Orientation |
| --- | --- | --- | --- | --- |
| *CSH1* | -33 to -19 | **AAAAAAAAAAACAGG** | 2.49e-05 | + |
| *PBR1* | -207 to -193 | **AAAACAAAAGGAAAG** | 4.10e-05 | + |
| *RBT5* | -316 to -302 | **CAAAACAAAACAAAG** | 2.29e-05 | - |
| *WH11* | -53 to -39 | **AAAAAAAAAGGAAGG** | 1.43e-04 | - |
| *TEC1* | -538 to -524 | **GAAAAAAAAATAAAG** | 5.72e-05 | + |
| EAP1 | -163 to -149 | **AAAAAAAAATAAAGG** | 2.98e-05 | - |
| *PGA10* | -140 to -126 | **AGAAGAAAATGAAAG** | 2.84e-05 | - |
| *LSP1* | -197 to -183 | **AAAAAAAAAGGAAGG** | 2.02e-05 | - |
| *PHR1* | -834 to -820 | **AAAAAAAAAACCAAG** | 4.10e-05 | + |
| *PHR2* | -145 to -131 | **AAAAAAAAAAGAAAG** | 2.02e-06 | - |
| *SUN41* | -671 to -657 | **AAAAACAAAACAAAG** | 4.10e-05 | - |
| *Orf19.2077* | -94 to -80 | **AAAAAAAAAGGAAAG** | 6.99e-05 | + |
| *CIT1* | -82 to -68 | **AAAAAAAAGTGAAAG** | 9.86e-05 | - |
| STE2 | -786 to -772 | **AAAAAAAAAACCAAC** | 1.85e-04 | - |
| *CEK2* | -200 to -186 | **AAAAAAATAAAAAAA** | 3.43e-04 | + |
| *SST2* | -323 to -309 | **AAAGAAACCAAAAAA** | 4.61e-04 | - |
| *RBT1* | -706 to -692 | **AGAAAAAACAGAAAG** | 2.66e-05 | + |
| Consensus | | **AAAAAAAAAAGAAAG** |  |  |

1. *C. dubliniensis*:

| Gene | WPRE  position | WPRE | P value | Orientation |
| --- | --- | --- | --- | --- |
| *Cd36_03770* | -382 to -368 | **GTATAAGAAAGAAGG** | 2.38e-04 | + |
| *Cd36_05950* | -682 to -668 | **CAAAAAAGAAGAATT** | 1.72e-05 | + |
| *Cd36_40190* | -964 to -950 | **CAAGAAAAAAAAAAG** | 2.27e-04 | + |
| *Cd36_19680* | -53 to -39 | **GAAAAAAAAAGAATG** | 4.99e-05 | - |
| *Cd36_84480* | -784 to -770 | **TAAAAAAAAAGAAGA** | 2.36e-05 | + |
| *Cd36_23630* | -647 to -633 | **CAAAAAAAGAGAAAA** | 1.13e-05 | + |
| *Cd36_40510* | -257 to -243 | **ACACAAAAATGAAAT** | 5.88e-04 | - |
| *Cd36_21030* | -392 to -378 | **AAAAAAAAAAGAATT** | 9.01e-06 | + |
| *Cd36_44230* | -820 to -806 | **AGAAAAAAAAAAGTG** | 4.59e-04 | - |
| *Cd36_00220* | -138 to -124 | **GAAAAAAAAAGTAAG** | 1.94e-04 | - |
| *Cd36_60800* | -168 to -154 | | **AAAAGAAGAAGAAGA** |  | | --- | --- | | 3.85e-05 | + |
| *Cd36_15470* | -74 to -60 | **AAAAAAAAAAGAAAG** | 9.19e-05 | + |
| *Cd36_28700* | -205 to -191 | **AAAAAAAAAAAATTG** | 7.50e-05 | + |
| *Cd36_32150* | -786 to -772 | **CAAAAAAAGAGAAAA** | 9.10e-06 | + |
| *Cd36_31320* | -226 to -212 | **AAAAAAAAAAGAATA** | 2.36e-05 | - |
| *Cd36_51980* | -378 to -364 | **AAAAAAAAAATAATG** | 5.96e-05 | + |
| *Cd36_43400* | -718 to -704 | **AAAAAAAAGAAAAAG** | 9.19e-06 | - |
| Consensus | | **AAAAAAAAAAGAAAG** |  |  |

1. *C. tropicalis*:

| Gene | WPRE  position | WPRE | P value | Orientation |
| --- | --- | --- | --- | --- |
| *CTRG_04744* | -646 to -632 | **CAAGTGACAAGAAAA** | 8.50e-05 | + |
| *CTRG_04251* | -542 to -528 | **AGAGAGGGAAAAAAA** | 3.24e-07 | - |
| *CTRG_00099* | -80 to -66 | **GGAGAGGAAAAAAAA** | 2.40e-06 | - |
| *CTRG_01660* | -39 to -25 | **CAACAAAAAACAAAA** | 8.02e-04 | + |
| *CTRG_02294* | -269 to -255 | **AAAGAAAGAAAAAAA** | 3.93e-05 | + |
| *CTRG_02074* | -736 to -722 | **GAAGTGACAGGAACA** | 8.98e-04 | - |
| *CTRG_00109* | -80 to -66 | **GGAGAGGGAAAAAAA** | 1.06e-07 | - |
| *CTRG_01890* | -57 to -43 | **AAAGAAGAAAAAAAA** | 2.63e-05 | - |
| *CTRG_03942* | -949 to -935 | **GAACAGGGAAAAAAA** | 6.18e-06 | - |
| *CTRG_04296* | -651 to -637 | **AAACACGAAAAAAAA** | 7.14e-05 | + |
| *CTRG_02944* | -940 to -926 | **AGAGAGAGAAGAAAA** | 3.46e-05 | - |
| *CTRG_01124* | -868 to -854 | **GAAAAAGAAATAAAA** | 1.02e-04 | - |
| *CTRG_00747* | -834 to -820 | **AAAGACAGAAAAAAA** | 3.95e-05 | - |
| *CTRG_00685* | -195 to -181 | **AGAGACAGAAAAAAA** | 3.13e-05 | - |
| *CTRG_00659* | -756 to -742 | **AAAGAGAAAACAGAA** | 4.25e-04 | + |
| *CTRG_03200* | -890 to -876 | **AAAGAAGGAAGAAAA** | 2.63e-05 | + |
| *CTRG_00477* | -674 to -660 | **GAAGAGGGAAAAAAA** | 5.41e-07 | + |
| Consensus | | **AAAGAGGGAAAAAAA** |  |  |
